# Phenotypes and gene expression profiles of *Saccharopolyspora erythraea *rifampicin-resistant (*rif*) mutants affected in erythromycin production

**DOI:** 10.1186/1475-2859-8-18

**Published:** 2009-03-30

**Authors:** Elisabetta Carata, Clelia Peano, Salvatore M Tredici, Francesco Ferrari, Adelfia Talà, Giorgio Corti, Silvio Bicciato, Gianluca De Bellis, Pietro Alifano

**Affiliations:** 1 Department of Biological and Environmental Sciences and Technologies, University of Salento, 73100 Lecce, Italy; 2 Institute for Biomedical Technologies, National Research Council, 20090 Segrate (Milan), Italy; 3 Department of Biology, University of Padova, 35121 Padova, Italy; 4 Department of Biomedical Sciences, University of Modena and Reggio Emilia, 41100 Modena, Italy

## Abstract

**Background:**

There is evidence from previous works that bacterial secondary metabolism may be stimulated by genetic manipulation of RNA polymerase (RNAP). In this study we have used rifampicin selection as a strategy to genetically improve the erythromycin producer *Saccharopolyspora erythraea*.

**Results:**

Spontaneous rifampicin-resistant (*rif*) mutants were isolated from the parental strain NRRL2338 and two *rif *mutations mapping within *rpoB*, S444F and Q426R, were characterized. With respect to the parental strain, S444F mutants exhibited higher respiratory performance and up to four-fold higher final erythromycin yields; in contrast, Q426R mutants were slow-growing, developmental-defective and severely impaired in erythromycin production. DNA microarray analysis demonstrated that these *rif *mutations deeply changed the transcriptional profile of *S. erythraea*. The expression of genes coding for key enzymes of carbon (and energy) and nitrogen central metabolism was dramatically altered in turn affecting the flux of metabolites through erythromycin feeder pathways. In particular, the valine catabolic pathway that supplies propionyl-CoA for biosynthesis of the erythromycin precursor 6-deoxyerythronolide B was strongly up-regulated in the S444F mutants, while the expression of the biosynthetic gene cluster of erythromycin (*ery*) was not significantly affected. In contrast, the *ery *cluster was down-regulated (<2-fold) in the Q426R mutants. These strains also exhibited an impressive stimulation of the nitrogen regulon, which may contribute to lower erythromycin yields as erythromycin production was strongly inhibited by ammonium.

**Conclusion:**

Rifampicin selection is a simple and reliable tool to investigate novel links between primary and secondary metabolism and morphological differentiation in *S. erythraea *and to improve erythromycin production. At the same time genome-wide analysis of expression profiles using DNA microarrays allowed information to be gained about the mechanisms underlying the stimulatory/inhibitory effects of the *rif *mutations on erythromycin production.

## Background

Actinomycetes are used for fermentative production of a wide range of bioactive molecules including antibiotics, anticancer agents and immune-suppressants. A crucial point of this process is that these microorganisms must often be genetically improved for higher production before they can be used in the industry. Historically, strain improvement has been carried out by multiple rounds of random mutagenesis and selection in close association with process improvements to optimize large-scale industrial fermentations [1]. Since the late 1970s, the availability of molecular genetics tools and useful information about the biosynthetic pathways and genetic control for most of secondary metabolites of commercial interest has opened the way for improving strains by rational engineering [2,3]. More recently, these rational strain improvement strategies benefit of the support of genomic, transcriptomic, proteomic, and metabolomic technologies [4-11].

The erythromycin fermentation is a classic antibiotic fermentation that has been improved by the traditional mutate-and-screen method over the past 50 years. Erythromycin biosynthesis in the mycelial actinomycete, *Saccharopolyspora erythraea*, has been widely studied as a model system for antibiotic production [12-15], and erythromycin and its semi-synthetic derivatives are widely used in the clinic; therefore, improved producers are still highly sought after.

Erythromycin A is made by a three-stage pathway [16]: assembly of the 14-membered macrolactone 6-deoxyerythronolide B (6DEB) from one propionyl-CoA and six (2S)-methylmalonyl-CoA units followed by its hydroxylation to erythronolide B (EB), formation of the deoxysugars mycarose and desosamine from glucose and their addition to EB to make erythromycin D, and then C-12 hydroxylation and C-3" O-methylation of the latter compound to produce erythromycin A. The synthesis of 6DEB is catalyzed by multifunctional modular polyketide synthase with at least 28 distinct active sites, in a process similar to that of fatty-acid biosynthesis [17,18].

Extensive genetic studies have provided some insight into the genes involved in erythromycin biosynthesis [19,20]. The erythromycin gene cluster contains 20 genes arranged in four major polycistronic units [21]. Evidence for regulatory genes has been missing for a long time hampering efforts to enhance erythromycin production other than by medium manipulation, random mutagenesis and selection. In recent times, the availability of the entire genome sequence of *S. erythraea *has opened the possibility of defining the mechanisms by which erythromycin is controlled by using global approaches [22,23]. Very recently, these approaches have led to the discovery that BldD, a key developmental regulator in actinomycetes [24,25], regulates the synthesis of erythromycin [26]. Meanwhile, there is evidence that increasing the flux through feeder metabolic pathways strongly influences the erythromycin yields. This has been recently obtained by engineering the methylmalonyl-CoA metabolite node in *S. erythraea *and in *Aeromicrobium erythreum*, a non-filamentous erythromycin A producer [27-29].

The focus of this study was to explore the possibility to increase the erythromycin production by genetic manipulation of the RNA polymerase (RNAP) of *S. erythraea*. This working hypothesis relies on well-documented evidence that: i) ppGpp, the effector of the stringent response [30], triggers antibiotic production in streptomycetes [31-35]; ii) several rifampicin-resistance mutations (*rif*) in the RNAP beta chain confer ppGpp-independent biosynthesis of the pigmented actinorhodin (Act) and undecylprodigiosin (Red), methylenomycin, and calcium-dependent antibiotic (CDA) in the model actinomycete *Streptomyces coelicolor *A3(2) [36]; *rif *mutations also activate cryptic antibiotic biosynthesis in *Streptomyces lividans*, a fast-growing close relative of *S. coelicolor *A3(2), which produces less or no Act, Red and CDA despite the existence of all the required biosynthetic genes [37-41]. Mechanistically, it has been proposed that several *rif *mutations mimic binding of ppGpp to RNAP [36,40].

These premises prompted us to investigate: i) the role of the stringent response in the activation of erythromycin biosynthesis; ii) the utility of the *rif *screening to search for erythromycin over-producing mutants; iii) how transcriptional changes, analyzed at a whole genome level, could shed light on the molecular mechanisms underlying, on one hand, erythromycin over-production by *rif1 *mutants, and on the other the almost null antibiotic production by *rif6 *mutants.

## Results and Discussion

### Isolation and phenotypes of *S. erythraea rif *mutants

**Figure 1:**
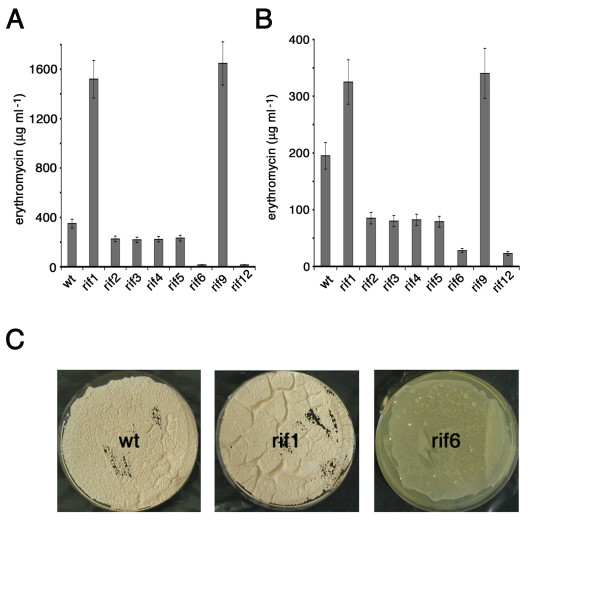
**Erythromycin production by *S. erythraea *NRRL2338 and derivative *rif *mutants on R3/1 or YS agar media**. (A-B) Strains were grown for 7 days on R3/1 (A) or YS (B) agar media and antibiotic production was evaluated by microbiological assays. Data are shown as mean ± standard deviation of triplicate samples in representative experiments. Similar results were obtained in three independent experiments. (C) Pictures of *S. erythraea *NRRL2338 and *rif *derivatives rif1 and rif6 after 7 days growth on R3/1 solid medium. Note in rif6 the severe defect in aerial mycelium and spore formation.

Twenty spontaneous *S. erythraea rif *mutants were isolated on YS agar containing 36 μg ml^-1 ^of rifampicin. By rapid screening on YS and R3/1 agar media, two of these mutants exhibited a clear higher-producing phenotype (rif1 and rif9), two mutants were severely impaired in the ability to produce erythromycin (rif6 and rif12), while the remaining others showed antibiotic yields slightly higher or lower with respect to that of the wild type strain (data not shown). Eight *rif *mutants, rif1, rif9, rif6, rif12, rif2, rif3, rif4 and rif5 were further analyzed (Figure 1). After a 7-day (168 h) incubation at 30°C on complex R3/1 solid medium, rif1 and rif9 produced an amount of antibiotic more than 4-fold higher than that of the parental strain (Figure 1A). In contrast, antibiotic production by rif6 and rif12 was barely detectable by microbiological assay. Antibiotic yields by rif2, rif3, rif4 and rif5 were almost identical and slightly lower than that of the wild type.

Rif6 and rif12 mutants were conditionally defective in antibiotic production. Indeed, their defect was less apparent when the strains were grown on complex YS solid medium (Figure 1B and data not shown). It should be noted, however, that the erythromycin production by the wild type strain and by rif1 and rif9 mutants was significantly lower on YS than on R3/1. Phenotypical analysis demonstrated that rif6 and rif12 were severely defective in aerial mycelium development and spore formation on R3/1 (Figure 1C and data not shown). In contrast, on the same medium rif1 and rif9 exhibited luxurious growth and more abundant spores with respect to the wild type.

**Figure 2:**
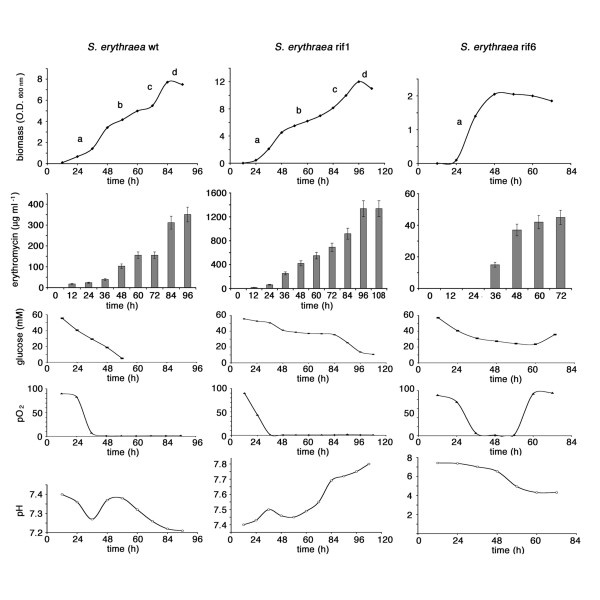
**Bioreactor cultures of *S. erythraea *NRRL2338 and derivative *rif *mutants**. Biomass, erythromycin production, glucose concentration, pO_2 _and pH were evaluated as described in the Materials and Methods section.

The different erythromycin production by the wild type, the rif1 and the rif6 mutants was confirmed in bioreactor experiments using R3/1 broth under standard batch-culture conditions (Figure 2). Consistently with previous findings [23] four distinct phases of the growth curve could be detected in both the wild type and the rif1 mutant: an initial period of rapid growth lasting until 36 h (Phase **a**), followed by a period of growth slowdown until 72 h (Phase **b**), a second period of rapid growth from 72 to 84 h (Phase **c**) before entering the stationary phase (Phase **d**). Erythromycin production was detectable after 12 h and then paralleled the biomass increase up to 96 h. In contrast, the rif6 mutant prematurely ceased growth after 48 h (Phase **a**) with a marked acidification of the medium and very low final antibiotic yields.

### Genotypic characterization of the *S. erythraea rif *mutants

In prokaryotes more than 90% of rifampicin-resistant isolates have missense mutations, deletions or insertions in the 81-bp rifampicin resistance-determining region (RRDR) of the *rpoB *gene [42-44]. To determine the location and nature of the *S. erythraea rif *mutations, this region was amplified by PCR from the wild type and mutant strains and subjected to nucleotide sequencing. The sequence analysis demonstrated: i) a C to T transition at position 1333 of the nucleotide sequence of the coding region of *S. erythraea rpoB *in the rif1 and rif9 mutants; ii) a C to T transition at position 1326 in the rif2, rif3, rif4 and rif5 mutants; iii) an A to G transition at position 1279 in the rif6 and rif12 mutant strains.

**Figure 3:**
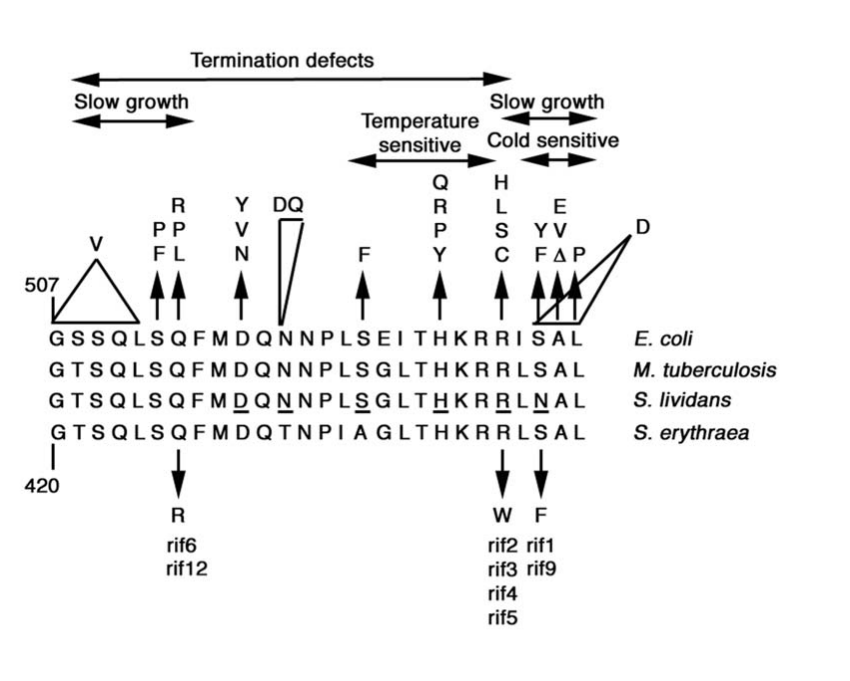
**Location and nature of the *S. erythraea *rif mutations**. Alignment of deduced amino acid sequence of rifampicin resistance-determining region (RRDR) of *E. coli*, *Mycobacterium tuberculosis*, *S. lividans *and *S. erythraea *with location of: i) the *rif *(*rpoB*) mutations more frequently associated with rifampicin-resistance in *E. coli *and their relative phenotypes (upper part of the panel); ii) the amino acid residues (underlined in the middle part of the panel) that, when mutated, are responsible for activation of cryptic antibiotic biosynthesis in *S. lividans*; iii) the missenses of the *S. erythraea *rif1-rif6 mutants.

These mutations resulted in the following missenses: S444F (rif1 and rif9), R442W (rif2, rif3, rif4 and rif5), Q426R (rif6 and rif12) affecting three conserved residues in prokaryotes, which are frequently involved in rifampicin resistance (Figure 3). R442W and S444F were of particular interest because it has been demonstrated that missense mutations R440H or R440C and N442Y affecting corresponding residues in *S. lividans rpoB *activate antibiotic biosynthesis in this microorganism (see Background).

Stimulation of erythromycin production in the rif1 and rif9 mutants (harboring the S444F missense) was thus consistent with the results obtained in *S. lividans*. Indeed, phenylalanine and tyrosine (replacing, respectively, a serine residue in *S. erythraea *and an asparagine residue in *S. lividans*) are structurally similar amino acids. In contrast, the R442W missense was not functionally equivalent to R440H or R440C in *S. lividans *and thus failed to stimulate erythromycin production. Interestingly, the missense Q426R in the slow-growing and developmental-defective rif6 and rif12 mutants affected a glutamine residue that, when mutated to proline, leucine or arginine (Figure 3) was responsible for transcription termination defects and slow growth in *E. coli *[42-46].

### Microarray analysis of the transcriptome of the *S. erythraea rif *mutants

To gain information about the mechanisms underlying the stimulatory/inhibitory effects of the *rif *mutations on erythromycin production, genome-wide analysis of expression profiles using DNA microarrays was performed. To this purpose, the wild type strain and the rif1 (S444F) and rif6 (Q426R) mutants were grown in shake-flasks containing R3/1 medium to either phase **a **or phase **b **of the growth curve (i.e. 24 h for wt and rif1 and 48 h for rif6; Figure 2). RNA samples were extracted from two independent cultures, processed and hybridized to custom made GeneChips containing DNA oligonucleotide probes corresponding to all the predicted *S. erythaea *ORFs.

Expression data of the wild type, rif1 and rif6 mutants during growth phases **a **and **b **were compared using Significance Analysis of Microarray (SAM) multiclass analysis. Setting the q-value threshold at 1% allowed identifying 198 and 270 differentially expressed genes (DEG) among wild type, rif1 and rif6 strains in phases **a **and **b**, respectively (see Additional file 1, Table S1 and Additional file 2, Figure S1). Among the 198 DEG characterizing the phase **a **[23], the five most represented gene-functional classes showing a significant enrichment were: II.6-Posttranslational modification, protein turnover, chaperone; II.8-RNA processing and modification; II.12-Translation, ribosomal structure and biogenesis; III.5-Energy production and conversion; III.8-Nucleotide transport and metabolism. The gene-functional classes showing a significant depletion were: I.3-Cell wall/membrane/envelope biogenesis, III.2-Carbohydrate transport and metabolism.

Among the 270 DEG characterizing the phase **b **the only functional category showing a significant enrichment was II.12-Translation, ribosomal structure and biogenesis, while a significant depletion was evidenced only for the functional category III.10-Secondary metabolites biosynthesis, transport catabolism.

**Figure 4:**
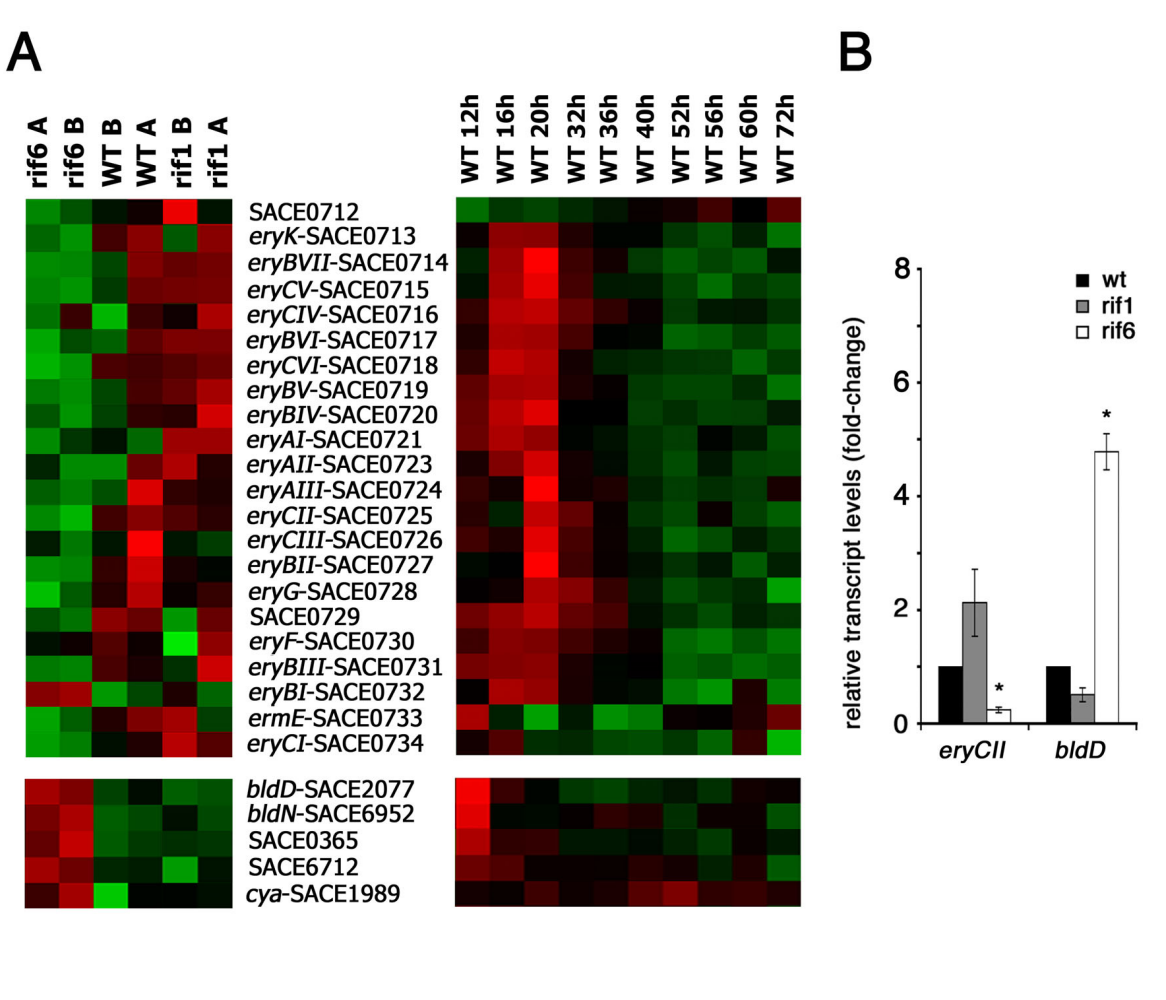
**Transcript analysis of the *ery *cluster and regulatory genes**. (A) Microarray analysis. Visualization by dChip of the expression of the *ery *cluster (upper panels) and regulatory genes (lower panels) during the time course of the wild type strain (right panels) and during phase **a **in the wild type and the rif1 and rif6 mutants (left panels). Red = up-regulation; Green = down-regulation. (B). Semi-quantitative analysis of *eryCII*- and *bldD*-specific transcripts by RT real-time PCR. The RNAs were extracted from *S. erythraea *NRRL2338 and *rif *derivatives rif1 and rif6 grown in R3/1 medium up to phase **a**. Results were normalized to 16S rRNA levels. Transcript levels of *S. erythraea *NRRL2338 were arbitrarily given a value of 1. Data are shown as mean ± standard deviation from three independent experiments, each with triplicate samples, using distinct cDNA preparations for each RNA sample. The Student's t-test was used for statistical analysis. Statistically significant differences between values from *S. erythraea *NRRL2338 and *rif *mutants (asterisks) are declared at a p value < 0.05.

For further analysis and discussion, we focused our attention on the 198 DEG of the growth phase **a **(see Additional file 1, Tables S2, S3, S4 and S5) when expression of erythromycin biosynthetic genes was maximal in the wild type strain (Figure 4A, right panel). Indeed, any comparison among the two mutant strains and the wild type during the phase **b **(see Additional file 1, Tables S6, S7, S8, S9 and S10) was considered uninformative because of the severe growth phenotype of rif6. The 198 DEG were grouped into four clusters (Cluster 1 to 4).

### Cluster 1

**Figure 5:**
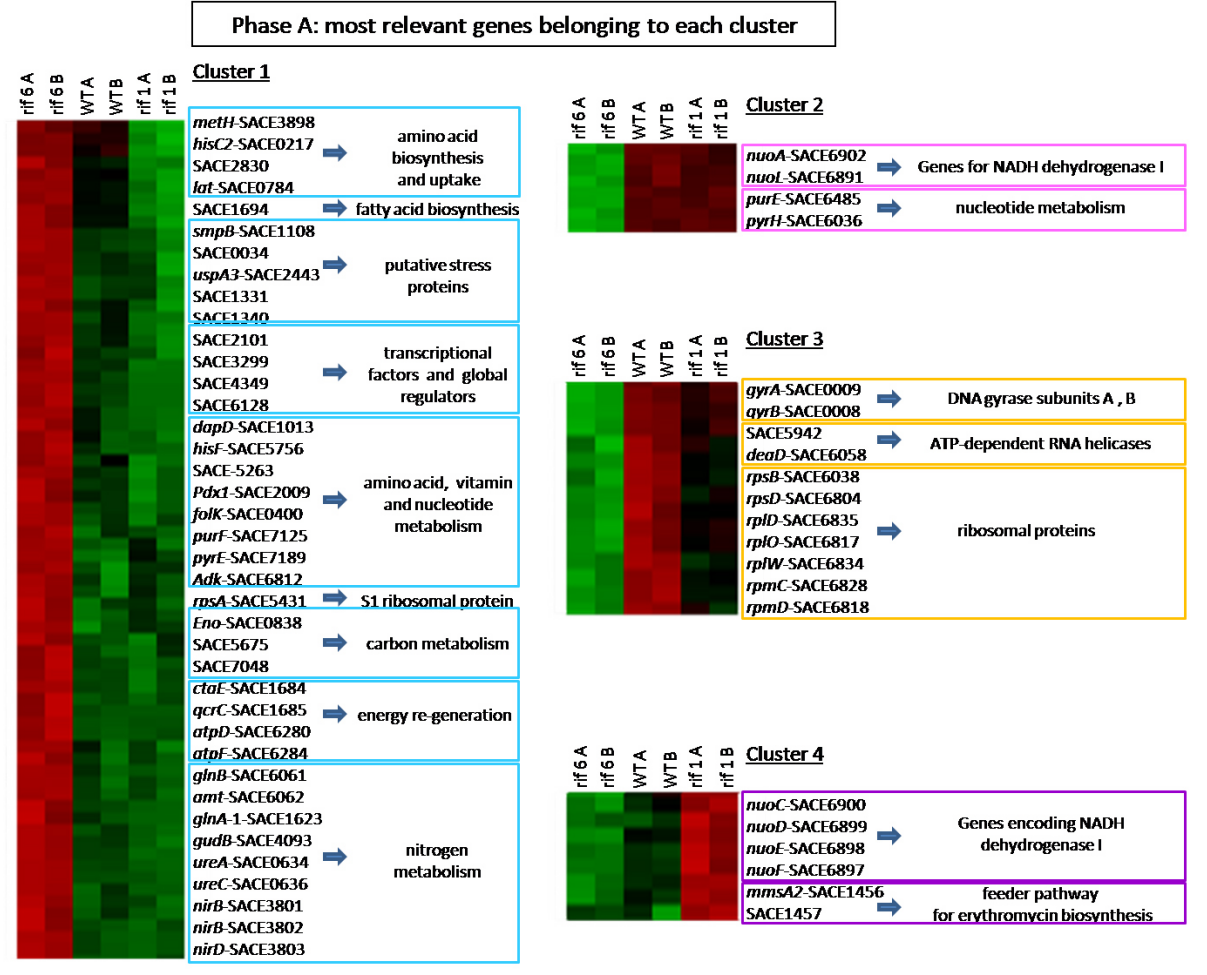
**Microarray analysis of the most relevant DEGs**. Visualization by dChip of the most relevant genes belonging to each of the four clusters formed by phase **a**-DEGs. Red = up-regulation; Green = down-regulation.

This cluster is the largest one and comprises 122 genes that were up-regulated in rif6 and not affected in the rif1 compared to the wild type (Figure 5, left panel). This cluster includes genes involved in amino acid biosynthesis (*metH*, *hisC2*, *lat *corresponding to SACE 3898, SACE 0217 and SACE 0784, respectively) and uptake (SACE 2830) and in fatty acid biosynthesis (SACE 1694 coding for putative long-chain fatty acid ligase). Cluster 1 includes also genes coding for putative stress proteins (*smpB *[SACE 1108], SACE 0034, *uspA3 *[SACE 2443], SACE 1331 and SACE 1340), transcriptional factors (SACE 2101 coding for the omega subunit of RNAP) and global transcriptional regulators (SACE 3299, SACE 4349, SACE 6128), and genes involved in amino acid (*dapD *[SACE 1013], *hisF *[SACE 5756], SACE 5263), vitamin (*pdx1 *[SACE 2009], *folK *[SACE 0400]) and nucleotide metabolism (*purF *[SACE 7125], *pyrE *[SACE 7189], *adk *[SACE 6812]). Other very relevant genes belonging to this cluster are: *rpsA *(SACE 5431) coding for S1, the largest ribosomal protein, and genes encoding proteins involved in carbon metabolism (*eno *[SACE 0838] coding for the phosphopyruvate hydratase, SACE 5675 coding for the pyruvate dehydrogenase complex, E1 component, beta subunit, and SACE 7048 encoding the 2,5-diketo-D-gluconic acid reductase) and energy re-generation (*ctaE *[SACE 1684] coding for the cytochrome C oxidase subunit III, *qcrC *[SACE 1685] coding for the cytochrome C mono- and di-heme variants, *atpD *[SACE 6280] and *atpF *[SACE 6284] coding for the ATP synthase beta and B chains, respectively).

This cluster includes also many genes involved in nitrogen metabolism: *glnB *(SACE 6061), encoding the nitrogen regulatory protein PII, *amt *(SACE 6062), coding for an ammonium transporter, *glnA-1 *(SACE 1623), coding for the glutamine synthetase, *gudB *(SACE 4093), coding for the NAD-specific glutamate dehydrogenase, *ureA *(SACE 0634) and *ureC *(SACE 0636), coding for alpha and gamma subunits of the urease respectively, *nirB *(SACE 3801, SACE 3802) and *nirD *(SACE 3803), encoding the assimilatory nitrite reductase large and small subunits respectively, and *narK *coding for a nitrite extrusion protein.

Up-regulation of the nitrogen regulon, which in actinomycetes includes genes involved in ammonium assimilation and supply [47,48], is noteworthy because demonstrates that the erythromycin production is strongly inhibited by ammonium in the wild type strain (see Additional file 2, Figure S2) and is supported by the evidence that, in *Amycolatopsis mediterranei*, GlnR links rifamycin biosynthesis to nitrogen metabolism [49].

### Cluster 2

This cluster comprises 36 genes that were down-regulated in the rif6 mutant and not affected in the rif1 compared to the wild type (Figure 5, right up-panel). The most important members of this cluster are genes encoding NADH dehydrogenase I (*nuoA *[SACE 6902] and *nioL *[SACE 6891]), and two genes involved in nucleotide metabolism (*purE *[SACE 6485] and *pyrH *[SACE 6036]). More interestingly, all genes of the *ery *cluster except *eryBI *(SACE 0732) showed a similar gene expression profile, being down-regulated in the rif6 mutant and not significantly affected in the rif1 compared to the wild type (Figure 4A, left panel). In contrast, *eryBI*, which is transcribed monocistronically with the transcript start site facing toward that of *eryE *[21], exhibited an opposite behavior, e.g. being up-regulated in the rif6 mutant and not significantly affected in the rif1 compared to the wild type. It is worthwhile noting that, although none of the genes of the *ery *cluster had a statistically significant differential expression at the stringent q-value threshold of 1%, half of them (i.e., *eryCVI*, *eryCI*, *eryBI*, *eryCV*, *eryBV*, *eryCII*, *eryBII*, *eryG*, *eryBVI*, *eryBVII*) were statistically significant at the still stringent q-value threshold of 5%. Down-regulation of *eryCII *in the rif6 mutant was supported by reverse transcriptase real-time PCR analysis (Figure 4B).

Unexpectedly, *bldD*, coding for a key developmental regulator that seems to regulate the *ery *cluster positively, exhibited an opposite gene expression pattern with respect to most of *ery *genes. In particular, similarly to the genes of cluster 1, *bldD *was up-regulated in the rif6 and not significantly affected in the rif1 compared to the wild type (Figure 4A, left panel and Figure 4B). It should be noted, however, that *bldD *mRNA levels may not parallel BldD protein levels because the regulation of *bldD *expression in actinomycetes is very complex and involves different mechanisms including transcription repression by BldD [26,50] and developmental stage-dependent proteolysis [51]. Thus, it is possible that the increased *bldD *mRNA levels may reflect an enhanced BldD proteolysis in the rif6 mutant. This hypothesis is supported by up-regulation, in this strain, of many genes which are known to be negatively regulated by BldD in streptomycetes [25] including *bldN *(SACE 6952), coding for a developmental regulator, *cya *(SACE 1989), coding for an adenylate cyclase, a two-component system response regulator (SACE 6712) and a HAD-superfamily subfamily IB, PSPase-like protein (SACE 0365) (Figure 4A, left panel). In addition to a possible effect on the expression of the *ery *cluster, the altered BldD-dependent regulation may also account for the "bald" phenotype of this mutant (Figure 1C). Future work will be aimed at verifying this hypothesis.

### Cluster 3

This cluster comprises 27 genes that were moderately down-regulated in the rif1 and strongly down-regulated in the rif6 compared to the expression levels into the wild type (Figure 5, right central-panel). This cluster includes genes coding for DNA gyrase subunits A and B (*gyrA *[SACE 0009] and *gyrB *[SACE 0008], respectively), ATP-dependent RNA helicases (SACE 5942 and *deaD *[SACE 6058]) and a large number of ribosomal proteins (*rpsB*, *rpsD*, *rplD*, *rplO*, *rplW*, *rpmC *and *rpmD *corresponding to SACE 6038, SACE 6804, SACE 6835, SACE 6817, SACE 6834, SACE 6828 and SACE 6818, respectively).

Apparently, down-regulation of genes encoding ribosomal proteins and up-regulation of genes involved in amino acid biosynthesis (cluster 1, see above) was suggestive for an ability of the *rif6 *mutations to induce a stringent phenotype as well as other well-characterized *rif *mutations (see Background). However, it should be pointed out that in *Corynebacterium glutamicum *up-regulation of ribosomal protein operons was also observed in ppGpp-defective strains following treatment with serine hydroxamate, a serine analogue that competitively binds to the seryl-tRNA synthetase and prevents the seryl-tRNA from being charged thus inducing the stringent response [52].

### Cluster 4

**Figure 6:**
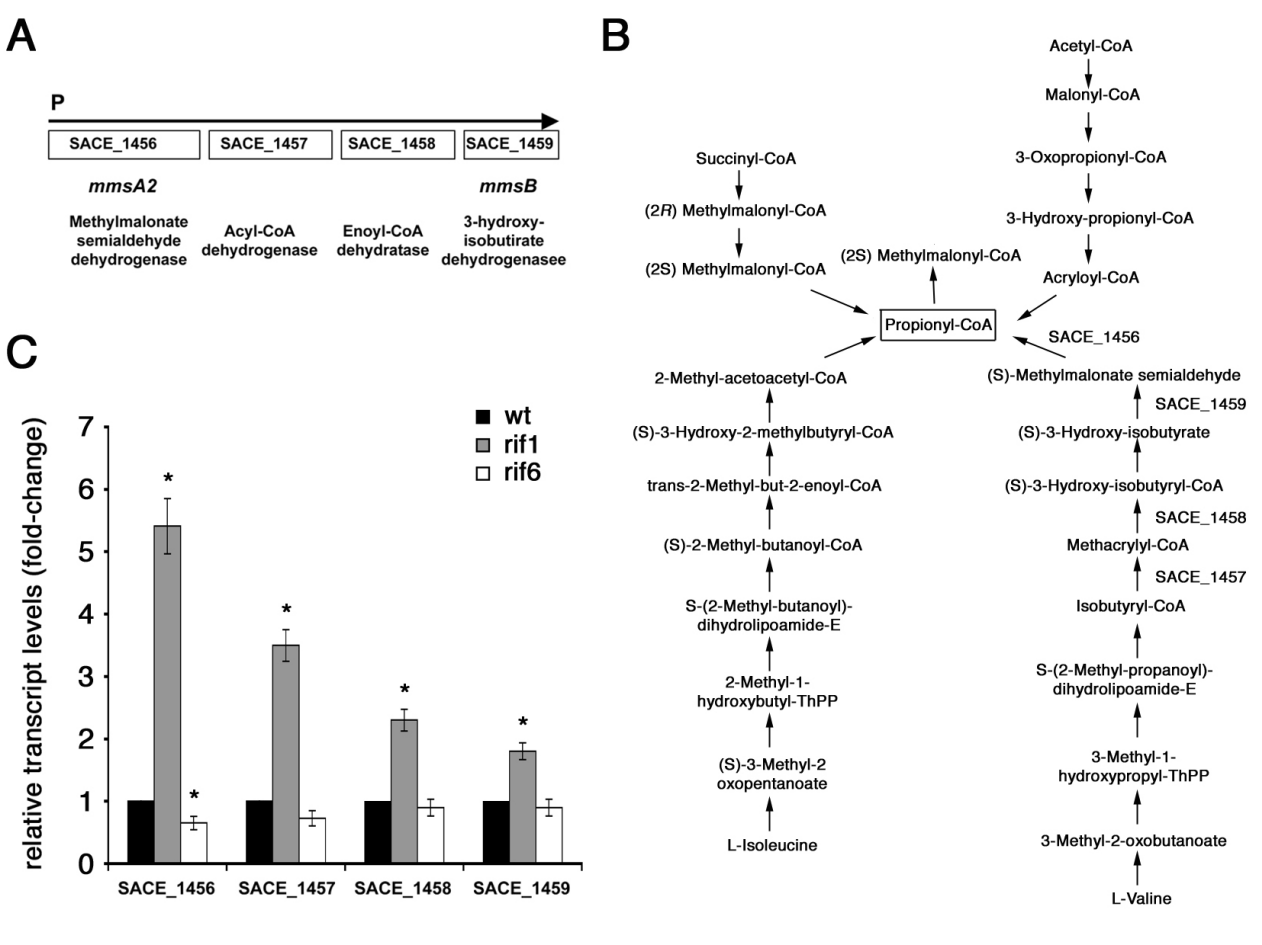
**Semi-quantitative analysis of the SACE 1456-SACE 1459-specific transcripts by RT real time PCR**. (A) Genetic map of the SACE 1456-SACE 1459 genetic cluster coding for enzymes involved in the valine catabolic pathway (B). (B) Possible metabolic pathways leading to propionyl-CoA and 2-methylmalonil-CoA, the two building blocks for biosynthesis of the erythromycin precursor 6-deoxyerythronolide B. (C) Semi-quantitative analysis of SACE 1456-SACE 1459-specific transcripts by RT real-time PCR. The RNAs were extracted from *S. erythraea *NRRL2338 and *rif *derivatives rif1 and rif6 grown in R3/1 medium up to phase **a**. Results were normalized to 16S rRNA levels. Transcript levels of *S. erythraea *NRRL2338 were arbitrarily given a value of 1. Data are shown as mean ± standard deviation from three independent experiments, each with triplicate samples, using distinct cDNA preparations for each RNA sample. The Student's t-test was used for statistical analysis. Statistically significant differences between values from *S. erythraea *NRRL2338 and *rif *mutants (asterisks) are declared at a p value < 0.05.

This cluster comprises 13 genes that were up-regulated in rif1, and down-regulated in the rif6 (Figure 5, right down-panel). The most important members of this cluster are genes encoding NADH dehydrogenase I (*nuoC *[SACE 6900], *nuoD *[SACE 6899], *nuoE *[SACE 6898], *nuoF *[SACE 6897]), and genes coding for key enzymes of a major feeder pathway of erythromycin biosynthesis (*mmsA2 *[SACE 1456] coding for methylmalonate semialdehyde dehydrogenase, SACE 1457 coding for acyl-CoA dehydrogenase-like activity). In particular SACE 1456 and SACE 1457 are the first two genes of a putative operon also including SACE 1458 (*echA9*) encoding putative enoyl-CoA hydratase/isomerase and SACE 1459 (*mmsB*) coding for 3-hydroxyisobutyrate dehydrogenase (Figure 6A).

As the *ery *cluster was found to be only slightly down-regulated in the rif6 and not significantly affected in the rif1, it is likely that the different yields of erythromycin in the rif1 and the rif6 mutants may be due, at least in part, to a differential activity of this major feeder pathway (Figure 6B). To validate the microarray analysis and obtain more quantitative data, the amounts of *mmsA2*- and *mmsB*-specific transcripts were measured by RT real-time PCR. The results confirmed the up-regulation of the cluster SACE 1456-SACE 1459 in the rif1 mutant (Figure 6C).

Moreover, the data analysis identified additional clusters of contiguous genes sharing similar transcriptional modulation. These groups of physically proximal genes contain, in most cases, members of known or putative operons showing similar expression profile (Additional file 2, Figure S3) including: i.) the *str *locus containing the S10-*spc*-alpha ribosomal protein operons [53]; ii.) the *rpsB-tsf-pyrH *operon coding for ribosomal protein S2, elongation factor Ts and UMP-kinase [54,55]; iii.) the *nuo *operon encoding the energy-generating NADH dehydrogenase complex I [54]; iv.) the *ctaE-qcrCA *gene locus coding for cytochrome c oxidase subunit III and for subunits of the ubiquinol c reductase including cytochrome cc and a Rieske Fe-S protein, respectively [56]; v.) the F0 F1 ATP synthase operon containing the eight genes *atpBEFHAGDC *[57]; vi.) the *ure *operon (*ureABCFGD*) coding for all subunits of the urease and its accessory proteins [58]; vii.) a putative *nar*/*nir *gene cluster coding for a nitrite extrusion protein (NarK), the catalytic subunit of the assimilatory nitrate reductase (NasA/NarB), the large (NirB) and small (NirD) subunits of NAD(P)H-nitrite reductase and two enzymes involved in the biosynthesis of siroheme (HemD and NirE/SirB), the prostetic group of nitrite reductase [59]; viii.) the *amt-glnB *operon coding for the ammonium transporter and the nitrogen regulon regulatory protein PII. Up-regulation of the *amt-glnB *operon in the rif6 mutant may be due to increased *glnR *(SACE 7101) mRNA levels. Indeed, there is evidence for a central role of GlnR in regulation of nitrogen metabolism in actinomycetes [48]. The expression profiles of several genes located in these operons were confirmed by RT real-time PCR (Additional file 2, Figure S4).

## Conclusion

This study demonstrates the usefulness of the *rif *screening as a tool to search for higher-producer strains and provides new information about the molecular mechanisms underlying the stimulatory effect of several *rif *mutations on bacterial secondary metabolism. The *rif *mutations deeply changed the transcriptional profile of *S. erythraea *suggesting that their effects on erythromycin biosynthesis go beyond the stringent response, as previously reported (see Introduction). In addition to specific effects on clusters of genes coding for secondary metabolites, the expression of genes coding for key enzymes of the carbon and nitrogen central metabolism was dramatically altered affecting, in turn, energy supply, growth rates and fluxes of metabolites through the erythromycin feeder pathways.

The *ery *cluster was found to be slightly down-regulated in the hypo-producing rif6 mutant possibly as a consequence of perturbed BldD-dependent regulation. In contrast, the expression of the *ery *cluster was slightly affected in the rif1 mutant. In this strain, the enhanced activity of an erythromycin feeder pathway may account for the hyper-producing phenotype.

The intimate connection between the erythromycin biosynthesis and the central metabolism is consistent with both genomic and expression data. In general, secondary metabolism is believed dispensable for survival, and most of gene clusters coding for secondary metabolites occupy non-core genomic regions and are maximally expressed during late growth phases. In contrast, the *ery *cluster maps in the core region of the *S. erythraea *chromosome, and is transcribed during the middle pseudo-exponential growth phase when the activities of the carbon and nitrogen central metabolic pathways are maximal [22,23,60]. As shown in figure 6B, these pathways are strictly connected to erythromycin biosynthesis. Indeed, assembly of the 14-membered macrolactone 6DEB requires one propionyl-CoA and six (2*S*)-methylmalonyl-CoA units.

The analysis of the hyper-producing mutant rif1 suggests that the valine catabolic pathway, which is strongly up-regulated in this strain, may be a major feeder pathway supplying propionyl-CoA. Propionyl-CoA is then transformed into (2*S*)-methylmalonyl-CoA biotin-dependent carboxylation that in *S. erythraea *may be accomplished by at least five isoenzymes [22,23]. On the other hand, there is experimental evidence that the erythromycin biosynthetic pathway is connected to the Krebs cycle via the methylmalonyl-CoA mutase, an adenosylcobalamin-dependent enzyme that catalyzes the reversible isomerization of (2*R*)-methylmalonyl-CoA and succinyl-CoA [27-29]. This connection may underlie the negative regulation of erythromycin production by ammonium in starch-based fermentations accounting, at least in part, for the phenotype of the hypo-producing mutant rif6 in which the nitrogen regulon, including genes for ammonium uptake and assimilation, was impressively up-regulated.

## Methods

### Bacterial strains and media

*S. erythraea *wild type strain NRRL2338 was a gift of S. Donadio (KtedoGen, Milano). This strain has been deposited at the American Type Culture Collection. The strain was stored in 1-ml cryotubes at -80°C as frozen mycelium in YS medium containing 15% glycerol at a biomass concentration of approximately 0.25 g dry cell weight (DCW) ml^-1^, or at -20°C as spores in 20% glycerol (in distilled water) at a title of approximately 5 × 10^8 ^ml^-1^.

The composition (per liter) of the complete media used in this study is reported in Table 1. When requested all media were agarized at a concentration of 1.8%.

**Table 1:** Composition of the media used in this study

**Medium**	**Composition (per liter)**	**pH**
**Complex**		
Medium 707	5 g peptone, 3 g yeast extract, 1 g MgSO_4_·7 H_2_O	pH 7.0
Seed medium (SM)	4 g peptone, 4 g yeast extract, 2 g KH_2_PO_4_, 4 g K_2_HPO_4_, 0.5 g MgSO_4_7H_2_0, 10 g glucose	pH 7.2
R3/1	5 g yeast extract, 0.1 g casamino acids, 3 g L-proline, 10 g MgCl_2_·6H_2_O, 4 g CaCl_2_·2H_2_O, 0.2 g K_2_SO_4_, 0.05 g KH_2_PO_4_, 5.6 g TES, 10 g glucose	pH 7.2
Medium 266 (YS)	2 g yeast extract, 10 g soluble starch	pH 7.3
OMY	40 g oatmeal, 1 g yeast extract	pH 6.8–7.0
Nutrient broth	3 g beef extract, 5 g tryptone, 15 g NaCl	pH 7.2
		
**Chemically defined**		
MM-101	7 g NH_4_Cl, 3 g KH_2_PO_4_, 7 g K_2_HPO_4_, 0.25 g MgSO_4_·7 H_2_O, 0.0138 g CaCl_2_·2 H_2_O, 10 g glucose, 2 ml trace solution element (TSE)^a^	pH 6.9
MM-102	1 g NH_4_Cl, 1 g CaCO_3_, 0.5 g NaCl, 0.4 g MgSO_4_·7 H_2_O, 0.15 g KH_2_PO_4_, 0.35 g K_2_HPO_4_, 10 g glucose, 2 ml trace solution element (TSE)^a^	pH 6.9

^a^When indicated 1.0 g casamino acids (Difco, Detroit, Mich.) and/or 2 ml (per liter) trace solution element (TSE) were added. The TSE solution composition (per liter) was: 40 mg ZnCl_2_, 200 mg FeCl_3_·6 H_2_O, 10 mg CuCl_2_·2 H_2_O, 10 mg MnCl_2_·4 H_2_O, 10 mg Na_2_B_4_O_7_·10 H_2_O, 10 mg (NH_4_)_6_Mo_7_O_24_·4 H_2_O.

*Escherichia coli *strain DH5α was used in cloning procedures. This strain was grown in Luria Bertani (LB) medium. To allow plasmid selection, LB medium was supplemented with ampicillin (50 μg ml-1). The composition (per liter) of the nutrient broth agar in the microbiological assays with *Micrococcus luteus *tester strain was: 3 g beef extract, 5 g tryptone, 15 g NaCl, 15 g agar.

### Preparation of spores

Concentrated spore suspensions (5 × 10^8 ^ml^-1^) are crucial for purposes like starting reproducible cultures for physiological or fermentation studies. To prepare spores adapted to the conditions of liquid medium, spores were spread on the same medium with agar. Mycelium with spores was strongly attached to the surface agar, which made impossible to collect spores without agar traces. Therefore, the method of growing strains on cellophane disc was used [61]. The cellophane discs were sterilized in distilled water and then placed on agar, and the inoculum was spread on cellophane by glass stick. After two weeks, spores (control in microscope) were easily scraped from cellophane, and stored in 20% glycerol at -20°C.

### Growth conditions

For shake-flask experiments, spores in frozen aliquots were collected by centrifugation, re-suspended in medium 707 (for rehydration), and readily separated by vortexing. Individual aliquots (about 5 × 10^8 ^spores) were used to inoculate each 500 ml buffled Erlenmeyer flask containing 50 ml of the liquid media described above. Cultures were incubated at 30°C with shaking at 250 rpm. Bioreactor cultures were carried out by using Minifors mini-fermenters (Infors AG, Bottmingen, CH) that operated with a working volume of 1.5 l. Stirring was provided by Rushton-type impellors rotating at 250 rpm. Sterile air was supplied through a sparger. The bioreactors were equipped with pH electrode, pO2 electrode (polarographic), antifoam probe and Pt-100. Glucose concentration was monitored during fermentation by coupled glucose oxidase-peroxidase reaction using a commercial kit (distributed by Laboser srl).

### Erythromycin assays

Erythromycin production in solid media was assayed by bioassay. To this purpose, *S. erythraea *strains were grown in solid media (30 ml) in Petri dishes (8.5 cm). After desired time of cultivation, 1.6 cm (diameter) agar discs (with mycelium on the surface) were removed and placed into empty Petri dishes (diameter 8.5 cm) that were filled with soft nutrient agar that was seeded with *Micrococcus luteus*. Diameters of the zone of inhibition were measured after 2 days incubation at 37°C. Agar discs containing defined amounts of >95% pure erythromycin A (Sigma) were used as a reference. In liquid media, erythromycin was extracted and assayed by both microbiological assay and thin layer chromatography (TLC). Extraction of erythromycin from fermentation broths was performed as described [62]. TLC identification was carried out on silica-gel GF254 plates as previously described [63].

### DNA procedures

High molecular weight genomic DNA was extracted from *S. erythraea *strains grown in 50 ml of SM medium with shaking at 28°C for 5 days (120 h). After centrifugation, the mycelium was re-suspended in 10 ml SET buffer (75 mM NaCl, 25 mM EDTA, 20 mM Tris-Cl pH 7.5) and incubated in the presence of 5 mg ml^-1 ^lysozyme for 30' at 37°C. Samples were sonicated (Sonifer sonicator Model 250/240, Brain Ultrasonic Corporation) 3 times 30 sec, and incubated in the presence of 20 mg ml-1 Proteinase K and 1.2% sodium dodecyl sulfate (SDS) for 2 h at 55°C. Nucleic acids were extracted by fenol-chloroform:isoamylic alcohol (24:1) extraction according to standard procedure [64], and 15 μg ml-1 ribonuclease A was used to remove RNA. After fenol-chloroform:isoamylic alcohol (24:1) extraction and ethanol-precipitation, high molecular-weight DNA was collected by spooling using Shepherd's crooks [64].

DNA fragments were isolated through acrylamide slab gels and recovered by electro-elution as described [64]. Oligonucleotides used as primer in the PCR reactions are listed in Table 2. The amplification reactions consisted of 30 cycles including 1 min of denaturation at 94°C, 1 min of annealing at 55°C and 1–2 min of extension at 72°C. They were carried out in a Perkin Elmer Cetus DNA Thermal Cycler 480.

**Table 2:** Oligonucleotides used in RT real-time PCR experiments

**Name**	**Sequence**	**Target gene**	**Amplicon length** **(bp)**
16Suniv-1	5'-CAGCAGCCGCGGTAATAC-3'	16S rRNA	409
16Suniv-2	5'-CCGTCAATTCCTTTGAGTTT-3'		
SACE1456 for	5'-GCGGCTGGCCGAGCTGTTCATC-3'	SACE1456	172
SACE1456 rev	5'-GTGGGCGGCGGCCGTCGAGTAG-3'		
SACE1457 for	5'-GATGTCTACGTCGTGATGGCCAG-3'	SACE1457	184
SACE1457 rev	5'-CGAGCCGGTGCGTGGCGGGCAC-3'		
SACE1458 for	5'-GCTTTGCGCGGGCGGCGACATCC-3'	SACE1458	186
SACE1458 rev	5'-CCGTGCGCGGTGACACCGACG-3'		
SACE1459 for	5'-CGGCCAGGTCACCAAGATGTGC-3'	SACE1459	167
SACE1459 rev	5'-CCGGGCAGTTGGTGGTCAGCG-3'		
SACE0636 for	5'-CAACCCGACCCGCCCGCACAC-3'	SACE0636	107
SACE0636 rev	5'-CGGCGAAGGCGAGGTCCAC-3'		
SACE1684 for	5'-CCTCCCGTTCACGATCATCC-3'	SACE1684	140
SACE1684 rev	5'-GCCCCAGCACGAAGACCGTTC-3'		
SACE3802 for	5'-AGCCTCGGCCGCGGCCACGTCC-3'	SACE3802	102
SACE3802 rev	5'-CACCGAGTACGTGCCGTTGCGC-3'		
SACE6038 for	5'-GGCCTACGACTTCGTCAAG-3'	SACE6038	125
SACE6038 rev	5'-GGTTGACGAAGGGCATGC-3'		
SACE6062 for	5'-GTCGTGGGCGTGCTCTGG-3'	SACE6062	109
SACE6062 rev	5'-CCTGTCCGAGCCCGAAGAAC-3'		
SACE6280 for	5'-CAAGGCGCCGTCCTTCGACCAG-3'	SACE6280	112
SACE6280 rev	5'-GAACAGACCGATCTTGCCGCC-3'		
SACE6818 for	5'-GCACAAGGGTCTGGTCGG-3'	SACE6818	116
SACE6818 rev	5'-CCAGGCCACGCACGTCAGG-3'		
SACE6899 for	5'-GGGCGAGACGATCGTCAAGGCCC-3'	SACE6899	133
SACE6899 rev	5'-GTGCAGCGGCGCGAGGTAGTCC-3'		
SACE6902 for	5'-GTCCCGCTGGTGCTGATG-3'	SACE6902	100
SACE6902 rev	5'-CGTTGGCGCGGTTGTACC-3'		
SACE7101 for	5'-AGGAGGTCTGGGGCTACGACTTCTTCG-3'	SACE7101	110
SACE7101 rev	5'-CACGGTGCCGATGGAGTCGTAC-3'		
bldD for	5'-GGCCGAGAAGGTGGGCCCGCTG-3'	*bldD*	136
bldD rev	5'-CCGGGCGTCATGTCGTAGATG-3'		
eryCII for	5'-GACCCTTACCCGATGCTGCTG-3'	*eryCII*	156
eryCII rev	5'-GGTGAACGCGGGGTCGTCGAG-3'		

DNA sequencing was performed as a service by MWG Biotech. DNA similarity searches were carried out using BLAST at NCBI [65]. Sequence alignments were performed with Clustal W at EBI [66].

### RNA extraction, microarray and reverse transcriptase real-time PCR experiments

For each strain and time point, total RNA was extracted from mycelium pellets deriving from 1-ml culture samples using the GeneElute™ total RNA Purification Kit (SIGMA), recovering it in 50 μl of Elution Solution. After extraction RNAs were quantified with a NanoDrop spectrophotometer (NanoDrop Technologies) and analyzed by capillary electrophoresis on an Agilent Bioanalyzer (Agilent).

The RNA samples showing an RNA Integrity Number (i.e., the quality parameter calculated by the Bioanalyzer software) higher than 7 were processed for microarray hybridization, following the instructions of "Target Labeling for Prokaryotic GeneChip^® ^Antisense Arrays" (Affymetrix Prokaryotic gene Expression Manual). The protocol consists in cDNA synthesis by reverse transcription (starting with 10 μg RNA), followed by cDNA fragmentation with DNase I and labeling with Terminal Deoxynucleotidyl Transferase. The labeled cDNAs were then hybridized for 16 h at 50°C on individual *S. erythraea *GeneChips. After hybridization, GeneChips were washed and stained with streptavidin-conjugated phycoerythrin by using the Fluidic Station FS450 (Affymetrix) according to the ProkGE-WS2v3_450 Protocol. Fluorescent images of the microarrays were acquired using a GeneChip Scanner 3000 (Affymetrix). All Chip images and files have been deposited in the GEO (Gene Expression Omnibus) [67] repository (accession number: GSE12017).

The *S. erythraea *GeneChip was previously described in Peano et al. [23]. The microarray, targeting the whole set of *S. erythraea *genes, is composed of 25-mer oligonucleotide probes organized in 7060 probe-sets. The vast majority of probe-sets (i.e. 6494) are composed of at least 11 probes pairs (Perfect Match/MisMatch); among these, 29 probe-sets, targeting genes longer than 5000 bp, include from 13 to 86 probe pairs; the remaining 566 probe-sets are instead composed of less than 11 probe pairs.

Quantitative analysis of *eryCII*, *bldD*, SACE 0636, 1456–1459, 1684, 3802, 6038, 6062, 6280, 6818, 6899, 6902 and 7101 specific transcripts, normalized to 16S rRNA, was performed by reverse transcriptase (RT) real-time PCR. Total RNAs (1 μg) from *S. erythraea *NRRL2338 and several *rif *derivatives grown in R3/1 medium up to phase **a **(Figure 2) were reverse-transcribed by using random hexamer (2.5 μM) with Superscript RT (Invitrogen). About 0.1–1% of each RT reaction was used to run real-time PCR on a SmartCycler System (Cepheid) with SYBR^® ^Green JumpStart Taq ReadyMix (Sigma-Aldrich) and the primer pairs indicated in Table 2. Real-time PCR samples were run in triplicate. The real-time PCR conditions were: 10 min initial denaturation at 94°C, 30 sec at 94°C, 30 sec at 55°C, 30 sec at 72°C for 35 cycles; detection of PCR products was performed at 83°C.

### Data Analysis

The quality of the raw data obtained from microarray hybridization was assessed by MAS5.0 (Microarray Suite/Software, Affymetrix) control parameters after a global scaling at a target intensity of 100. Control parameters, as well as box plot of raw intensities, indicated the overall high quality of the data set and the absence of any outlying sample. Probe level data was converted to expression values using the Robust Multi-array Average (RMA) procedure [68]. Briefly, PM values (Perfect Match) were background-adjusted, normalized using quantile normalization, and log transformed.

Significance Analysis of Microarray (SAM) has been applied to detect differentially expressed genes.

SAM was introduced by Tusher [69] as a statistical technique for finding significant genes in microarrays while controlling the False Discovery Rate (FDR). SAM computes a statistic for each gene, measuring the strength of the relationship between gene expression and the response variable (e.g. the strain types, the growth phases, etc.). It uses repeated permutations of the data to determine if the expression level of any genes is significantly related to the response. The cutoff for significance is determined controlling the q-value, i.e. the lowest False Discovery Rate at which a gene is called significant [70]. Similarly to the p-value, the q-value measures how significant a gene is differentially expressed in the context of a large number of genes.

Hierarchical clustering and Eisen's maps were used to group modulated genes and samples in the software package dChip [71]. Before clustering, the expression values for a gene across all samples were standardized and these standardized values were used to calculate correlations between genes and samples and served as the basis for merging nodes. In the hierarchical agglomerative clustering Pearson correlation coefficient and centroid were used as distance metric and linkage method, respectively.

## Competing interests

The authors declare that they have no competing interests.

## Authors' contributions

EC isolated the *rif *mutants and carried out phenotypic analyses. CP performed the microarray experiments, participated in data analysis and drafted the manuscript. SMT carried out erythromycin assays. FF performed the data analysis, participated in the manuscript preparation. AT carried out genotypic analysis of the *rif *mutants and RT real-time PCR experiments. GC participated in data analysis and manuscript preparation. SB performed the data analysis, participated in manuscript preparation. PA and GDB conceived the study and participated in its design, coordination and manuscript preparation. All authors read and approved the final manuscript.

## Supplementary Material

Additional file 1 Functional categories significantly enriched or depleted in the 198 phase **a **and 270 phase **b **differentially expressed genes (DEGs) (Table S1). Phase **a**: 122 differentially expressed genes belonging to cluster 1 (up-regulated in rif6) (Table S2). Phase **a**: 36 differentially expressed genes belonging to cluster 2 (down-regulated in rif6) (Table S3). Phase **a**: 27 differentially expressed genes belonging to cluster 3 (strongly down-regulated in rif 6, moderately down-regulated in rif1) (Table S4). Phase **a**: 13 differentially expressed genes belonging to cluster 4 (up-regulated in rif1, down-regulated in rif 6) (Table S5). Phase **b**: 60 differentially expressed genes belonging to cluster 1 (up-regulated in rif1) (Table S6). Phase **b**: 8 differentially expressed genes belonging to cluster 2 (up-regulated in rif1 and rif 6) (Table S7). Phase **b**: 22 differentially expressed genes belonging to cluster 3 (up-regulated in rif6) (Table S8). Phase **b**: 63 differentially expressed genes belonging to cluster 4 (down-regulated in rif1) (Table S9). Phase **b**: 117 differentially expressed genes belonging to cluster 5 (down-regulated in rif 1 and rif 6) (Table S10). Click here for file

Additional file 2 Microarray analysis: sample dendrogram and Eisen's map of the 198 phase a and of the 270 phase b differentially expressed genes. (Figure S1). Effects of ammonium on *S. erythraea *pigmentation and erythromycin production in solid media (Figure S2). Members of known or putative operons showing similar expression profile (Figure S3). Semi-quantitative analysis of transcripts of genes located in known or putative operons by RT real time PCR (Figure S4). Click here for file
