# Supplementary material for: Phenotypes and gene expression profiles of Saccharopolyspora erythraea rifampicin-resistant (rif) mutants affected in erythromycin production
Source: Microb Cell Fact. 2009 Mar 30;8:18. doi: 10.1186/1475-2859-8-18 (PMC2667423; doi:10.1186/1475-2859-8-18)
Supplement: Additional file 1 — Functional categories significantly enriched or depleted in the 198 phase a and 270 phase b differentially expressed genes (DEGs) (Table S1). Phase a: 122 differentially expressed genes belonging to cluster 1 (up-regulated in rif6) (Table S2). Phase a: 36 differentially expressed genes belonging to cluster 2 (down-regulated in rif6) (Table S3). Phase a: 27 differentially expressed genes belonging to cluster 3 (strongly down-regulated in rif 6, moderately down-regulated in rif1) (Table S4). Phase a: 13 differentially expressed genes belonging to cluster 4 (up-regulated in rif1, down-regulated in rif 6) (Table S5). Phase b: 60 differentially expressed genes belonging to cluster 1 (up-regulated in rif1) (Table S6). Phase b: 8 differentially expressed genes belonging to cluster 2 (up-regulated in rif1 and rif 6) (Table S7). Phase b: 22 differentially expressed genes belonging to cluster 3 (up-regulated in rif6) (Table S8). Phase b: 63 differentially expressed genes belonging to cluster 4 (down-regulated in rif1) (Table S9). Phase b: 117 differentially expressed genes belonging to cluster 5 (down-regulated in rif 1 and rif 6) (Table S10). [file 1475-2859-8-18-S1.pdf]

Table S1. Functional categories significantly enriched or depleted in the 198 phase **a** and 270 phase **b** differentially expressed genes (DEGs)[a]

Functional categories	COG	Total probesets	Total %	DEG Phase A	Phase A %	DEG Phase B	Phase B %
**I. Cellular processes and signalling**							
Cell cycle control, cell division, chromosome partitioning	I.1	25	0.38	0	0.00	2	0.74
Cell motility	I.2	1	0.02	0	0.00	0	0.00
Cell wall/membrane/envelope biogenesis	*I.3*	*145*	*2.23*	*2*	*1.01*	5	1.85
Defense mechanisms	I.4	63	0.97	1	0.51	2	0.74
Intracellular trafficking, secretion, and vesicular transport	I.5	17	0.26	1	0.51	0	0.00
Posttranslational modification, protein turnover, chaperones	I.6	134	2.06	7	3.54	7	2.59
Signal transduction mechanisms	I.7	135	2.08	3	1.52	3	1.11
**II. Information storage and processing**							
Amino acid transport and metabolism	II.1	1	0.02	0	0.00	0	0.00
Cell wall/membrane/envelope biogenesis	II.2	1	0.02	0	0.00	0	0.00
Chromatin structure and dynamics	II.3	1	0.02	0	0.00	0	0.00
General function prediction only	II.4	21	0.32	1	0.51	0	0.00
Nucleotide transport and metabolism	II.5	5	0.08	0	0.00	0	0.00
**Posttranslational modification, protein turnover, chaperone**	**II.6**	**3**	**0.05**	**2**	**1.01**	0	0.00
Replication, recombination and repair	II.7	172	2.65	6	3.03	4	1.48
**RNA processing and modification**	**II.8**	**1**	**0.02**	**1**	**0.51**	0	0.00
Secondary metabolites biosynthesis, transport catabolism	II.9	3	0.05	0	0.00	0	0.00
Signal transduction mechanisms	II.10	50	0.77	1	0.51	0	0.00
Transcription	II.11	508	7.82	8	4.04	12	4.44
**Translation, ribosomal structure and biogenesis**	**II.12**	**175**	**2.69**	**12**	**6.06**	**15**	**5.56**
**III. Metabolism**							
Amino acid transport and metabolism	III.1	466	7.18	18	9.09	19	7.04
Carbohydrate transport and metabolism	*III.2*	*429*	*6.61*	*6*	*3.03*	21	7.78
Cell wall/membrane/envelope biogenesis	III.3	27	0.42	1	0.51	0	0.00
Coenzyme transport and metabolism	III.4	186	2.86	7	3.54	7	2.59
**Energy production and conversion**	**III.5**	**338**	**5.20**	**22**	**11.11**	27	10.00
Inorganic ion transport and metabolism	III.6	189	2.91	10	5.05	5	1.85
Lipid transport and metabolism	III.7	299	4.60	6	3.03	11	4.07
**Nucleotide transport and metabolism**	**III.8**	**90**	**1.39**	**10**	**5.05**	3	1.11
Posttranslational modification, protein turnover, chaperones	III.9	6	0.09	0	0.00	0	0.00
Secondary metabolites biosynthesis, transport catabolism	III.10	205	3.16	0	0.00	*2*	*0.74*
Signal transduction mechanisms	III.11	11	0.17	0	0.00	0	0.00
**IV. Poorly characterized**							
Function unknown	IV.1	2267	34.91	65	32.83	103	38.15
General function prediction only	IV.2	520	8.01	8	4.04	22	8.15

[a]The total of the ORFs targeted by the 6494 probe-sets analyzed, and the 198 phase **a** and 270 phase **b** differentially expressed genes, have been divided into functional categories and the enrichment or depletion of some of them was evidenced. For each functional category
BOLD-red = enrichment= % (DEG Phase A or DEG Phase B)>2*% (total probe-sets)
ITALIC-green = depletion= % (DEG Phase A or DEG Phase B)<2*% (total probe-sets)

Table S2. Phase **a**: 122 differentially expressed genes belonging to cluster 1 (up-regulated in rif6).

**Probe-set ID**	**Functional category**	**Gene name and Product description**
SACE_1966	I.3	---, hypothetical protein
SACE_7138	I.3	---, lysozyme M1 precursor
SACE_2146	I.5	---, preprotein translocase SecG subunit
SACE_0034	I.6	---, putative peptidyl-prolyl cis-trans isomerase
SACE_1108	I.6	smpB, SsrA-binding protein
SACE_2251	I.6	prcB, 20S proteasome beta-subunit precursor
SACE_3845	I.7	---, FHA domain containing protein
SACE_2443	I.7	uspA3, universal stress protein family
SACE_1682	I.7	---, putative two-component system response regulator
SACE_7101	II.10	glnR, transcriptional regulator
SACE_2101	II.11	---, DNA-directed RNA polymerase subunit omega
SACE_4349	II.11	---, transcriptional regulator, GntR family
SACE_4481	II.11	---, transcriptional regulator, ArsR family
SACE_3299	II.11	---, probable transcriptional regulator, MarR family
SACE_5148	II.11	---, GCN5-related N-acetyltransferase
SACE_1104	II.11	---, possible transcriptional regulator, PadR family
SACE_5431	II.12	rpsA, 30S ribosomal protein S1
SACE_6443	II.12	---, S30AE family protein
SACE_6690	II.4	---, putative serine/threonine protein kinase
SACE_0638	II.6	ureG, urease accessory protein
SACE_1292	II.8	ornA, oligoribonuclease (3'->5' exoribonuclease)
SACE_0784	III.1	lat, L-lysine-epsilon aminotransferase
SACE_1013	III.1	dapD, 2,3,4,5-tetrahydropyridine-2,6-dicarboxylate N-succinyltransferase
SACE_5756	III.1	hisF, imidazoleglycerol-phosphate synthase cyclase
SACE_1623	III.1	glnA-1, glutamine synthetase
SACE_0634	III.1	ureA, urease gamma subunit protein
SACE_5263	III.1	---, N-acetyl-gamma-glutamyl-phosphate reductase
SACE_0636	III.1	ureC, urease alpha subunit
SACE_6061	III.1	glnB, nitrogen regulatory protein PII
SACE_0217	III.1	hisC2, putative histidinol-phosphate aminotransferase
SACE_6995	III.1	---, N-succinyldiaminopimelate aminotransferase
SACE_3898	III.1	metH, putative 5-methyltetrahydrofolate:homocysteine S-methyltransferase
SACE_4093	III.1	gudB, NAD-specific glutamate dehydrogenase
SACE_1978	III.1	---, proline dehydrogenase
SACE_0838	III.2	eno, phosphopyruvate hydratase
SACE_6189	III.2	ebrB, multidrug resistance efflux protein
SACE_3945	III.2	---, metabolite transporter, MFS superfamily
SACE_0625	III.2	---, drug resistance transporter, Bcr/CflA family
SACE_1584	III.2	---, putative bifunctional protein (ribonuclease H/phosphoglycerate mutase)
SACE_4546	III.3	---, secreted protein
SACE_2009	III.4	pdx1, putative pyridoxine biosynthesis protein
SACE_0400	III.4	folK, 2-amino-4-hydroxy-6-hydroxymethyldihydropteridine pyrophosphokinase
SACE_1636	III.4	lipB, lipoate-protein ligase B
SACE_3804	III.4	---, uroporphyrinogen-III synthetase
SACE_6888	III.4	---, polyprenyl diphosphate synthase component
SACE_2830	III.5	---, probable Na(+)/glutamate:H(+) symporter
SACE_5674	III.5	---, pyruvate dehydrogenase complex dihydrolipoamide acetyltransferase
SACE_1684	III.5	ctaE, cytochrome c oxidase subunit III

SACE_1685	III.5	qcrC, cytochrome c mono-and diheme variants
SACE_5675	III.5	---, dehydrogenase complex, E1 component, beta subunit
SACE_1686	III.5	---, probable ubiquinol-cytochrome c reductase iron-sulfur subunit
SACE_6284	III.5	atpF, ATP synthase B chain
SACE_6280	III.5	atpD, ATP synthase beta chain
SACE_3674	III.5	mdh, malate dehydrogenase
SACE_0195	III.5	---, probable FAD linked oxidoreductase
SACE_3802	III.5	nirB, nitrite reductase (NAD(P)H) large subunit
SACE_3801	III.5	nirB, assimilatory nitrite reductase large subunit
SACE_3800	III.5	---, assimilatory nitrate reductase catalytic subunit
SACE_6062	III.6	amt, ammonium transporter
SACE_4973	III.6	---, ferritin family protein
SACE_3799	III.6	narK, nitrite extrusion protein
SACE_5235	III.6	---, TrkA-C domain protein
SACE_3803	III.6	nirD, nitrite reductase (NAD(P)H) small subunit
SACE_6539	III.6	---, probable multisubunit Na+:H+ antiporter MnhG subunit
SACE_1694	III.7	---, putative long-chain fatty acid ligase
SACE_7125	III.8	purF, amidophosphoribosyltransferase
SACE_6812	III.8	adk, adenylate kinase
SACE_7189	III.8	pyrE, orotate phosphoribosyltransferase
SACE_1461	III.8	---, guanine-specific ribonuclease N1 and T1
SACE_3612	III.8	add, adenosine deaminase
SACE_6727	III.8	---, allantoinase
SACE_1282	III.8	nrdB, ribonucleoside-diphosphate reductase, beta subunit
SACE_0335	IV.1	---, protein of unknown function DUF1469
SACE_5395	IV.1	---, hypothetical protein
SACE_0067	IV.1	---, hypothetical protein
SACE_0208	IV.1	---, hypothetical protein
SACE_2051	IV.1	---, hypothetical protein
SACE_6128	IV.1	---, putative transcriptional regulator, AsnC family
SACE_1577	IV.1	---, hypothetical protein
SACE_1955	IV.1	---, hypothetical protein
SACE_1394	IV.1	---, hypothetical protein
SACE_2077	IV.1	---, putative transcriptional regulator (DNA-binding protein)
SACE_0033	IV.1	---, hypothetical protein
SACE_0865	IV.1	---, hypothetical protein
SACE_1895	IV.1	---, transcriptional regulator, XRE family
SACE_0801	IV.1	---, transglycosylase-like protein
SACE_4318	IV.1	---, hypothetical protein
SACE_6787	IV.1	---, hypothetical protein
SACE_5834	IV.1	---, hypothetical protein
SACE_7176	IV.1	---, hypothetical protein
SACE_6957	IV.1	---, hypothetical protein
SACE_1697	IV.1	---, cyclase/dehydrase
SACE_2471	IV.1	---, hypothetical protein
SACE_1396	IV.1	---, hypothetical protein
SACE_5698	IV.1	---, hypothetical protein
SACE_1340	IV.1	---, DsbA oxidoreductase
SACE_1295	IV.1	---, hypothetical protein
SACE_6428	IV.1	---, biotin/lipoyl attachment
SACE_6926	IV.1	---, excisionase/Xis, DNA-binding
SACE_6556	IV.1	---, hypothetical protein
SACE_3805	IV.1	---, chalcone/stilbene synthase family protein
SACE_5214	IV.1	---, hypothetical protein

SACE_6241	IV.1	---, M protein
SACE_3841	IV.1	---, hypothetical protein
SACE_1460	IV.1	---, hypothetical protein
SACE_6574	IV.1	---, hypothetical protein
SACE_3736	IV.1	---, hypothetical protein
SACE_6608	IV.1	---, regulator
SACE_7071	IV.1	---, hypothetical protein
SACE_0081	IV.1	---, hypothetical protein
SACE_2475	IV.1	---, multicopper oxidase type 1
SACE_6397	IV.1	---, DGPF domain protein
SACE_2554	IV.1	---, possible transcriptional regulator
SACE_6426	IV.1	whiB1, putative transcriptional regulator (WhiB family)
SACE_5515	IV.1	---, hypothetical protein
SACE_6989	IV.1	tolA, membrane spanning protein
SACE_1754	IV.2	hflX, GTP-binding protein
SACE_7048	IV.2	---, 2,5-diketo-D-gluconic acid reductase
SACE_3559	IV.2	---, pyridoxamine 5'-phosphate oxidase-related,FMN-binding protein
SACE_0428	IV.2	---, antibiotic biosynthesis monooxygenase
SACE_1331	IV.2	glbO, hemoglobin-like, oxygen carrier
SACE_5733	IV.2	---, possible glycosyltransferase

Table S3. Phase **a**: 36 differentially expressed genes belonging to cluster 2 (down-regulated in rif6).

Probe-set ID	Functional category	Gene name and Product description
SACE_4615	I.4	---, putative ATP-binding component of an ABC transport protein
SACE_0341	II.11	ptlR, AraC-family transcriptional regulator
SACE_0542	II.11	---, transcriptional regulator, XRE family
SACE_2691	II.12	---, methionyl-tRNA formyltransferase
SACE_5243	II.7	nudF, ADP-ribose pyrophosphatase
SACE_2336	II.7	---, DNA polymerase III alpha subunit (fragment)
SACE_0848	III.1	---, oligopeptide ABC transporter ATP-binding protein
SACE_0847	III.1	---, oligopeptide ABC transporter ATP-binding protein
SACE_6157	III.1	ilvC, ketol-acid reductoisomerase
SACE_0508	III.4	thiG, thiamine synthesis, thiazole moiety
SACE_6902	III.5	nuoA, NADH dehydrogenase I chain A
SACE_6891	III.5	nuoL, NADH dehydrogenase I chain L
SACE_3507	III.5	---, probable CoB--CoM heterodisulfide reductase
SACE_1549	III.6	---, hypothetical protein
SACE_7096	III.6	pstB, putative phosphate transport ATP-binding protein
SACE_7106	III.6	cysA3, thiosulfate sulfurtransferase
SACE_0982	III.6	---, ABC transporter integral membrane protein
SACE_0440	III.7	ispF, 2-C-methyl-D-erythritol 2,4-cyclodiphosphate synthase
SACE_0337	III.7	acsA, acetyl-coenzyme A synthetase
SACE_4132	III.7	---, acyl carrier protein
SACE_6485	III.8	purE, phosphoribosylaminoimidazole carboxylase catalytic subunit
SACE_6036	III.8	pyrH, uridylate kinase
SACE_0874	IV.1	---, hypothetical protein
SACE_7080	IV.1	---, hypothetical protein
SACE_6471	IV.1	vrrB, hypervariable Bacillus group-specific protein
SACE_4465	IV.1	---, hypothetical protein
SACE_0197	IV.1	---, hypothetical protein
SACE_5903	IV.1	---, hypothetical protein
SACE_4561	IV.1	---, FAD dependent oxidoreductase
SACE_2972	IV.1	---, hypothetical protein
SACE_6652	IV.1	---, protein of unknown function DUF606
SACE_1101	IV.1	---, hypothetical protein
SACE_0256	IV.1	---, hypothetical protein
SACE_1735	IV.1	---, hypothetical protein
SACE_1131	IV.1	---, hypothetical protein
SACE_3626	IV.2	---, alpha/beta hydrolase fold

Table S4. Phase **a**: 27 differentially expressed genes belonging to cluster 3 (strongly down-regulated in rif 6, moderately down-regulated in rif1).

Probe-set ID	Functional category	Gene name and Product description
SACE_7210	I.6	dnaK, 70 kD heat shock protein (molecular chaperone)
SACE_5971	I.6	---, secreted trypsin-like serine protease
SACE_1359	I.6	tig, putative cell division trigger factor
SACE_5683	I.6	---, DsbA-like thioredoxin domain protein
SACE_6828	II.12	rpmC, 50S ribosomal protein L29
SACE_6818	II.12	rpmD, 50S ribosomal protein L30
SACE_5914	II.12	---, putative guanosine pentaphosphate synthetase and polyribonucleotide nucleotidyltransferase
SACE_6804	II.12	rpsD, 30S ribosomal protein S4
SACE_6835	II.12	rplD, 50S ribosomal protein L4
SACE_6834	II.12	rplW, 50S ribosomal protein L23
SACE_6038	II.12	rpsB, 30S ribosomal protein S2
SACE_6817	II.12	rplO, 50S ribosomal protein L15
SACE_6300	II.12	argS, arginyl-tRNA synthetase
SACE_6058	II.7	deaD, ATP-dependent RNA helicase
SACE_0009	II.7	gyrA, DNA gyrase subunit A
SACE_0008	II.7	gyrB, DNA gyrase subunit B
SACE_5942	II.7	---, ATP-dependent RNA helicase
SACE_2160	III.2	tkt, transketolase
SACE_1837	III.4	hemG, protoporphyrinogen oxidase
SACE_6585	III.5	sdhB, succinate dehydrogenase iron-sulfur protein
SACE_7143	III.8	purQ, phosphoribosylformylglycinamidine synthase I
SACE_3214	IV.1	---, hypothetical protein
SACE_6975	IV.1	---, ErfK/YbiS/YcfS/YnhG
SACE_1219	IV.1	---, transcriptional regulator, XRE family
SACE_5747	IV.1	---, hypothetical protein
SACE_1448	IV.1	---, transcriptional regulator, XRE family
SACE_2657	IV.1	---, hypothetical protein

Table S5. Phase **a**: 13 differentially expressed genes belonging to cluster 4 (up-regulated in rif1, down-regulated in rif 6).

Probe-set ID	Functional category	Gene name and Product description
SACE_2524	II.6	ureG, urease accessory protein
SACE_1987	III.1	---, amino acid permease-associated region
SACE_2526	III.1	ureC, urease alpha subunit
SACE_6900	III.5	nuoC, putative NADH dehydrogenase chain C
SACE_6897	III.5	nuoF, NADH dehydrogenase I chain F
SACE_6898	III.5	nuoE, putative NADH dehydrogenase chain E
SACE_6899	III.5	nuoD, NADH dehydrogenase I chain D
SACE_1456	III.5	mmsA2, methylmalonate-semialdehyde dehydrogenase (acylating)
SACE_3052	III.7	---, medium-chain acyl-CoA ligase
SACE_1457	III.7	---, acyl-CoA dehydrogenase-like
SACE_1553	IV.1	---, hypothetical protein
SACE_0177	IV.1	---, transcriptional regulator, XRE family
SACE_1550	IV.2	---, LamB/YcsF

Table S6. Phase **b**: 60 differentially expressed genes belonging to cluster 1 (up-regulated in rif1).

Probe-set ID	Functional category	Gene name and Product description
SACE_5921	I.4	dinF, DNA-damage-inducible protein F
SACE_1844	II.11	---, transcriptional regulator, XRE family
SACE_0016	II.11	---, transcriptional regulator, LuxR family
SACE_1609	II.11	helZ, probable helicase, Snf2/Rad54 family
SACE_6589	II.11	ydeS, similar to transcriptional regulator (TetR/AcrR family)
SACE_3836	II.11	---, acetyltransferase
SACE_2964	II.11	---, transcriptional regulator, GntR family
SACE_5885	II.11	---, putative TetR family transcriptional regulator
SACE_2113	II.12	fmt, methionyl-tRNA formyltransferase
SACE_3651	II.7	tnpA, Tn5045 transposase
SACE_1757	II.7	---, IS1533 transposase
SACE_0327	II.7	dnaN, DNA polymerase III beta subunit
SACE_5586	III.1	---, Zn-binding alcohol dehydrogenase
SACE_3404	III.1	---, serine/threonine dehydratase
SACE_5755	III.1	hisI, putative phosphoribosyl-AMP cyclohydrolase
SACE_4556	III.2	---, HpcH/HpaI aldolase
SACE_6531	III.2	eryA, putative erythritol kinase protein
SACE_4685	III.4	bioF, 8-amino-7-oxononanoate synthase
SACE_4394	III.4	menG, S-adenosylmethionine: 2-demethylmenaquinone methyltransferase protein
SACE_5965	III.4	chlI, putative magnesium-chelatase subunit
SACE_2788	III.5	---, monooxygenase, NtaA/SnaA/SoxA family
SACE_2405	III.5	---, probable pyruvate dehydrogenase, E2 component,dihydrolipoamide acetyltransferase
SACE_6645	III.6	---, phosphonate-binding protein
SACE_3153	III.7	---, AMP-dependent synthetase and ligase
SACE_5538	III.7	fabF, 3-oxoacyl-[acyl-carrier-protein] synthase
SACE_4705	III.7	---, short-chain dehydrogenase/reductase SDR
SACE_3857	III.7	fadD36, acyl-CoA synthase
SACE_4456	IV.1	---, hypothetical protein
SACE_2528	IV.1	---, hypothetical protein
SACE_1800	IV.1	---, hypothetical protein
SACE_2731	IV.1	---, hypothetical protein
SACE_7006	IV.1	---, secreted protein
SACE_1128	IV.1	---, hypothetical protein
SACE_2856	IV.1	---, possible secreted protein
SACE_3324	IV.1	---, hypothetical protein
SACE_0550	IV.1	---, hypothetical protein
SACE_1211	IV.1	---, hypothetical protein
SACE_0961	IV.1	---, possible suppressor protein DnaK
SACE_1345	IV.1	---, hypothetical protein
SACE_2570	IV.1	---, hypothetical protein
SACE_4528	IV.1	---, hypothetical protein
SACE_3121	IV.1	---, hypothetical protein
SACE_4426	IV.1	---, hypothetical protein
SACE_6164	IV.1	---, hypothetical protein
SACE_3987	IV.1	---, hypothetical protein
SACE_5911	IV.1	---, tetratricopeptide TPR_2 repeat protein
SACE_5418	IV.1	---, hypothetical protein

SACE_3368	IV.1	---, methylated-DNA-(protein)-cysteine S-methyltransferase
SACE_1888	IV.1	---, putative conserved alanine rich lipoprotein LppW
SACE_1663	IV.1	---, exo-alpha-sialidase
SACE_1141	IV.1	---, transcriptional regulator, XRE family
SACE_6577	IV.2	---, putative sugar ABC transporter integral membrane protein
SACE_5333	IV.2	---, putative oxidoreductase
SACE_4107	IV.2	---, putative helicase
SACE_6744	IV.2	blaB2, metallo-beta-lactamase
SACE_2505	IV.2	---, peptidase M50
SACE_7050	IV.2	---, putative ATP/GTP-binding protein
SACE_3929	IV.2	lipB, yfiP, lipase
SACE_6413	IV.2	---, methyltransferases-like
SACE_2148	IV.2	---, amidohydrolase family protein

Table S7. Phase **b**: 8 differentially expressed genes belonging to cluster 2 (up-regulated in rif1 and rif 6).

**Probe-set ID**	**Functional category**	**Gene name and Product description**
SACE_6340	I.6	---, trypsin-like serine protease
SACE_3811	III.5	acn, aconitate hydratase
SACE_3801	III.5	nirB, assimilatory nitrite reductase large subunit
SACE_5677	III.5	pdhD, dihydrolipoamide dehydrogenase
SACE_2477	III.5	---, phthalate 4,5-dioxygenase reductase subunit
SACE_0130	III.6	---, ferritin family protein
SACE_2305	IV.1	---, hypothetical protein
SACE_0474	IV.1	---, hypothetical protein

Table S8. Phase **b**: 22 differentially expressed genes belonging to cluster 3 (up-regulated in rif6).

**Probe-set ID**	**Functional category**	**Gene name and Product description**
SACE_1106	I.1	ftsE, cell division ATP-binding protein
SACE_2469	I.6	---, DsbA oxidoreductase
SACE_0527	I.6	groL2, 60 kDa chaperonin GroEL
SACE_6714	I.6	groEL1, 60 kD chaperonin cpn60
SACE_1414	II.12	rplU, 50S ribosomal protein L21
SACE_0636	III.1	ureC, urease alpha subunit
SACE_0634	III.1	ureA, urease gamma subunit protein
SACE_7201	III.2	---, integral membrane transport protein
SACE_0201	III.2	---, ABC-type transporter, permease component
SACE_7051	III.4	---, putative salicylate monooxygenase
SACE_3802	III.5	nirB, nitrite reductase (NAD(P)H) large subunit
SACE_6636	III.5	icd-2, isocitrate dehydrogenase, NADP-dependent
SACE_3800	III.5	---, assimilatory nitrate reductase catalytic subunit
SACE_6668	III.5	---, succinyl-coa synthetase alpha chain
SACE_6669	III.5	sucC, putative succinyl-CoA synthetase beta subunit
SACE_3674	III.5	mdh, malate dehydrogenase
SACE_3799	III.6	narK, nitrite extrusion protein
SACE_4231	IV.1	---, hypothetical protein
SACE_2475	IV.1	---, multicopper oxidase type 1
SACE_2471	IV.1	---, hypothetical protein
SACE_2474	IV.1	copC, putative integral membrane protein
SACE_0772	IV.1	---, hypothetical protein

Table S9. Phase **b**: 63 differentially expressed genes belonging to cluster 4 (down-regulated in rif1).

Probe-set ID	Functional category	Gene name and Product description
SACE_6130	I.3	ddl, D-alanylalanine synthetase
SACE_2525	I.6	ureF, urease accessroy protein
SACE_3845	I.7	---, FHA domain containing protein
SACE_1988	I.7	---, universal stress protein UspA
SACE_0408	II.11	---, putative Bordetella pertussis Bvg accessory factor family
SACE_6833	II.12	rplB, 50S ribosomal protein L2
SACE_6818	II.12	rpmD, 50S ribosomal protein L30
SACE_6828	II.12	rpmC, 50S ribosomal protein L29
SACE_6823	II.12	rpsN, 30S ribosomal protein S14
SACE_6808	II.12	infA, translation initiation factor IF1
SACE_6830	II.12	rpsC, 30S ribosomal protein S3
SACE_6832	II.12	rpsS, 30S ribosomal protein S19
SACE_6837	II.12	rpsJ, rpsX, 30S ribosomal protein S10
SACE_6806	II.12	rpsM, 30S ribosomal protein S13
SACE_6142	II.7	---, histone-like DNA binding protein
SACE_3254	III.1	---, amino acid permease-associated region
SACE_5669	III.1	---, amino acid transporter, APC family
SACE_0718	III.10	eryCVI, TDP-desosamine-N-dimethyltransferase
SACE_1047	III.2	---, ABC sugar transporter, permease component
SACE_5203	III.2	iolF, yxdF, inositol transport protein
SACE_1945	III.2	---, metabolite transporter, MFS superfamily
SACE_5454	III.2	---, multiple sugar transport system permease protein
SACE_2544	III.2	proP, proline/betaine transporter
SACE_0345	III.2	---, anion/cation symporter, MFS superfamily
SACE_5950	III.4	cobK, precorrin-6A reductase
SACE_6889	III.5	nuoN, NADH dehydrogenase I chain N
SACE_1678	III.5	ctaC, putative cytochrome c oxidase chain II
SACE_0939	III.7	---, 4-hydroxy-3-methylbut-2-enyl diphosphate reductase
SACE_3287	III.8	---, transmembrane transport protein
SACE_6486	III.8	purK, phosphoribosylaminoimidazole carboxylase ATPase subunit
SACE_2095	IV.1	---, hypothetical protein
SACE_6783	IV.1	---, hypothetical protein
SACE_6285	IV.1	atpE, ATP synthase C chain
SACE_4464	IV.1	---, hypothetical protein
SACE_1336	IV.1	---, hypothetical protein
SACE_5280	IV.1	rpmI, 50S ribosomal protein L35
SACE_5760	IV.1	---, hypothetical protein
SACE_4726	IV.1	---, hypothetical protein
SACE_0614	IV.1	---, hypothetical protein
SACE_6682	IV.1	---, DedA-like protein
SACE_1086	IV.1	---, hypothetical protein
SACE_7395	IV.1	ytjA, hypothetical protein
SACE_2088	IV.1	---, putative cytoplasmic protein
SACE_1703	IV.1	---, hypothetical protein
SACE_2491	IV.1	---, hypothetical protein
SACE_3743	IV.1	---, integral membrane protein

SACE_0039	IV.1	---, hypothetical protein
SACE_0213	IV.1	---, hypothetical protein
SACE_0212	IV.1	---, hypothetical protein
SACE_6807	IV.1	rpmJ, 50S ribosomal protein L36
SACE_5130	IV.1	---, DNA methylase
SACE_3200	IV.1	---, hypothetical protein
SACE_2539	IV.1	---, hypothetical protein
SACE_3204	IV.1	---, calcium binding protein
SACE_3237	IV.1	---, integral membrane protein
SACE_5652	IV.1	---, integral membrane protein
SACE_4834	IV.1	---, hypothetical protein
SACE_6374	IV.1	sti1, protease inhibitor precursor
SACE_7105	IV.1	---, hypothetical protein
SACE_6463	IV.2	---, integral membrane regulatory protein
SACE_3818	IV.2	---, protein of unknown function DUF81
SACE_1204	IV.2	---, Mov34/MPN/PAD-1
SACE_6102	IV.2	---, protein of unknown function DUF177

Table S10. Phase **b**: 117 differentially expressed genes belonging to cluster 5 (down-regulated in rif 1 and rif 6).

Probe-set ID	Functional category	Gene name and Product description
SACE_5830	I.1	---, cell division initiation protein
SACE_4937	I.3	---, mandelate racemase/starvation sensing protein
SACE_0675	I.3	---, lysozyme M1 precursor
SACE_0314	I.3	pon1, penicillin binding protein (class A)
SACE_7138	I.3	---, lysozyme M1 precursor
SACE_2202	I.4	---, carboxylpeptidase
SACE_1912	I.6	aprA, putative alkaline serine protease
SACE_2246	I.6	---, vesicle-fusing ATPase
SACE_6336	I.7	---, hypothetical protein
SACE_6393	II.11	ssfR, IclR-family transcriptional regulator
SACE_4934	II.11	---, AraC family transcriptional regulator
SACE_0525	II.11	---, cold-shock DNA-binding domain protein
SACE_7280	II.11	xylR, transcriptional regulator, ROK family
SACE_2008	II.12	fusA2, elongation factor EF-G
SACE_5297	II.12	---, hypothetical protein
SACE_6871	II.12	rplK, 50S ribosomal protein L11
SACE_6443	II.12	---, S30AE family protein
SACE_6885	III.1	---, amidinotransferase
SACE_2689	III.1	trpE, anthranilate synthase component I
SACE_5858	III.1	ilvD5, dihydroxy-acid dehydratase
SACE_0905	III.1	hpd3, 4-hydroxyphenylpyruvate dioxygenase
SACE_0784	III.1	lat, L-lysine-epsilon aminotransferase
SACE_4939	III.1	idnD, putative zinc-binding alcohol dehydrogenase
SACE_0845	III.1	---, putative peptide ABC transporter, permease component
SACE_5883	III.1	---, probable amino-acid acetyltransferase
SACE_3485	III.1	ureC, urease alpha subunit
SACE_1746	III.1	---, putative glutamate ABC transporter ATP-binding protein
SACE_1745	III.1	gluB1, putative glutamate binding protein
SACE_3873	III.1	dghA, glutamate dehydrogenase (NAD(P)+)
SACE_3033	III.10	---, putative peptide monooxygenase
SACE_1883	III.2	---, binding protein/LacI transcriptional regulator
SACE_4938	III.2	ydfJ, putative membrane transport protein
SACE_0949	III.2	---, carbohydrate kinase, FGGY
SACE_4940	III.2	---, fructuronate reductase
SACE_0941	III.2	rbsA, ribose transport ATP-binding protein
SACE_3487	III.2	---, general substrate transporter
SACE_0948	III.2	---, L-fucose isomerase, putative
SACE_7279	III.2	---, putative extracellular solute-binding lipoprotein
SACE_0943	III.2	---, binding protein/LacI transcriptional regulator
SACE_3717	III.2	---, integral membrane transport protein
SACE_6126	III.2	---, proline/betaine transporter, MFS family
SACE_4673	III.4	---, aminotransferase class III
SACE_3542	III.4	paaK, phenylacetate-CoA ligase
SACE_4754	III.5	acoA, acetoin dehydrogenase E1 component, alpha subunit
SACE_4260	III.5	lldP, lctP, L-lactate permease
SACE_4672	III.5	mmsA1, methylmalonate-semialdehyde dehydrogenase (acylating)

SACE_1170	III.5	sdhA1, succinate dehydrogenase flavoprotein subunit
SACE_3420	III.5	---, N5,N10-methylene-tetrahydromethanopterin reductase
SACE_1171	III.5	sdhB1, succinate dehydrogenase iron-sulfur protein
SACE_0536	III.5	---, carbon monoxide dehydrogenase small chain
SACE_4261	III.5	glcD, (S)-2-hydroxy-acid oxidase, subunit D
SACE_1449	III.5	aceA, isocitrate lyase
SACE_4755	III.5	acoB, acetoin dehydrogenase, beta subunit
SACE_1456	III.5	mmsA2, methylmalonate-semialdehyde dehydrogenase (acylating)
SACE_0537	III.5	---, carbon monoxide dehydrogenase large subunit
SACE_6895	III.5	nuoH, NADH dehydrogenase I chain H
SACE_3571	III.6	---, hypothetical protein
SACE_3034	III.6	---, possible ABC Fe(3+) transporter binding protein
SACE_5168	III.7	fadE10, acyl-CoA dehydrogenase
SACE_5343	III.7	---, AMP-dependent synthetase and ligase
SACE_1458	III.7	echA9, putative enoyl-CoA hydratase/isomerase
SACE_5382	III.7	fadE16, acyl-CoA dehydrogenase
SACE_5992	III.7	---, 1-hydroxy-2-methyl-2-(E)-butenyl 4-diphosphate synthase
SACE_0897	III.7	fadA3, acetyl-CoA acetyltransferase
SACE_6573	III.8	add, adenosine deaminase
SACE_4310	IV.1	---, hypothetical protein
SACE_2698	IV.1	---, putative regulatory protein
SACE_2918	IV.1	---, hypothetical protein
SACE_1392	IV.1	---, hypothetical protein
SACE_1399	IV.1	---, hypothetical protein
SACE_5747	IV.1	---, hypothetical protein
SACE_1168	IV.1	---, hypothetical protein
SACE_1397	IV.1	---, hypothetical protein
SACE_0176	IV.1	---, hypothetical protein
SACE_0767	IV.1	---, hypothetical protein
SACE_6639	IV.1	---, hypothetical protein
SACE_5363	IV.1	---, hypothetical protein
SACE_1103	IV.1	---, hypothetical protein
SACE_0177	IV.1	---, transcriptional regulator, XRE family
SACE_6381	IV.1	---, hypothetical protein
SACE_1398	IV.1	sti1, protease inhibitor precursor
SACE_1303	IV.1	---, probable myosin
SACE_0900	IV.1	---, putative antigen 34 kDa family
SACE_0037	IV.1	---, hypothetical protein
SACE_0696	IV.1	---, hypothetical protein
SACE_1054	IV.1	---, hypothetical protein
SACE_0917	IV.1	---, hypothetical protein
SACE_3976	IV.1	---, cyclic nucleotide-binding domain protein
SACE_6426	IV.1	whiB1, putative transcriptional regulator (WhiB family)
SACE_5738	IV.1	---, hypothetical protein
SACE_0372	IV.1	---, hypothetical protein
SACE_1393	IV.1	---, hypothetical protein
SACE_1528	IV.1	---, hypothetical protein
SACE_0033	IV.1	---, hypothetical protein
SACE_7221	IV.1	---, hypothetical protein
SACE_6519	IV.1	---, PE-PGRS family protein
SACE_0060	IV.1	---, hypothetical protein
SACE_7264	IV.1	---, hypothetical protein
SACE_3977	IV.1	---, terpene synthase, metal binding domain protein
SACE_3699	IV.1	---, hypothetical protein

SACE_3187	IV.1	---, terpene synthase, metal binding domain protein
SACE_0069	IV.1	---, regulator
SACE_7282	IV.1	---, secreted hydrolase
SACE_6338	IV.1	---, hypothetical protein
SACE_3372	IV.1	---, hypothetical protein
SACE_1097	IV.1	---, hypothetical protein
SACE_0226	IV.1	---, hypothetical protein
SACE_0801	IV.1	---, transglycosylase-like protein
SACE_3488	IV.2	---, CBS domain protein
SACE_6394	IV.2	---, protein of unknown function DUF59
SACE_3486	IV.2	---, exoenzymes regulatory protein AepA precursor
SACE_4756	IV.2	acoC, acetoin dehydrogenase, dihydrolipoamide acetyltransferase component
SACE_6395	IV.2	---, amidohydrolase 2
SACE_6396	IV.2	---, alcohol dehydrogenase, zinc-binding
SACE_6580	IV.2	---, ABC transporter substrate-binding protein
SACE_3417	IV.2	---, hydrolase
SACE_2699	IV.2	---, alpha/beta hydrolase fold
