# Supplementary material for: Phenotypes and gene expression profiles of Saccharopolyspora erythraea rifampicin-resistant (rif) mutants affected in erythromycin production
Source: Microb Cell Fact. 2009 Mar 30;8:18. doi: 10.1186/1475-2859-8-18 (PMC2667423; doi:10.1186/1475-2859-8-18)
Supplement: Additional file 2 — Microarray analysis: sample dendrogram and Eisen's map of the 198 phase a and of the 270 phase b differentially expressed genes. (Figure S1). Effects of ammonium on S. erythraea pigmentation and erythromycin production in solid media (Figure S2). Members of known or putative operons showing similar expression profile (Figure S3). Semi-quantitative analysis of transcripts of genes located in known or putative operons by RT real time PCR (Figure S4). [file 1475-2859-8-18-S2.pdf]

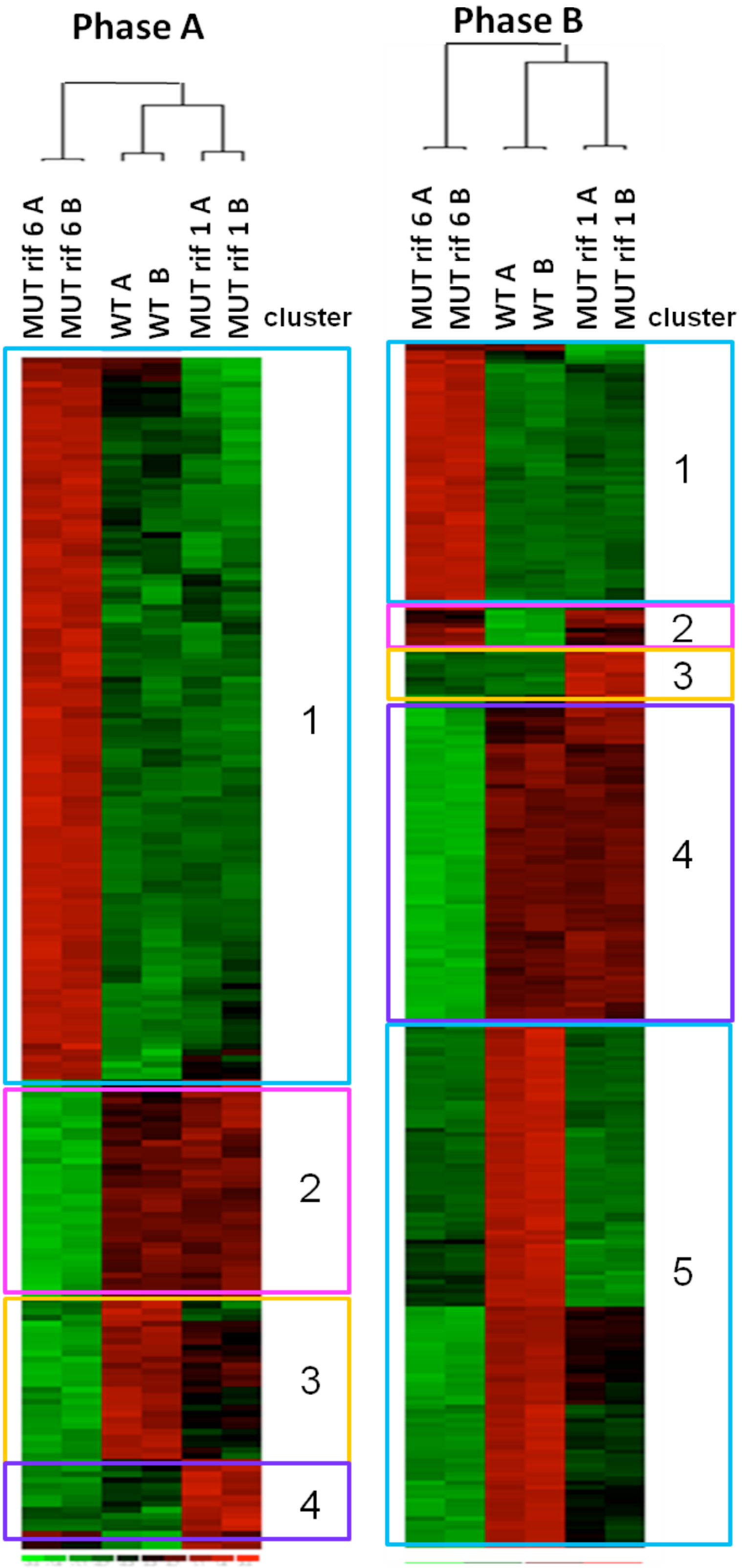

Figure S1

Microarray analysis. Sample dendrogram and Eisen's map of the 198 phase **a** and of the 270 phase **b** differentially expressed genes.

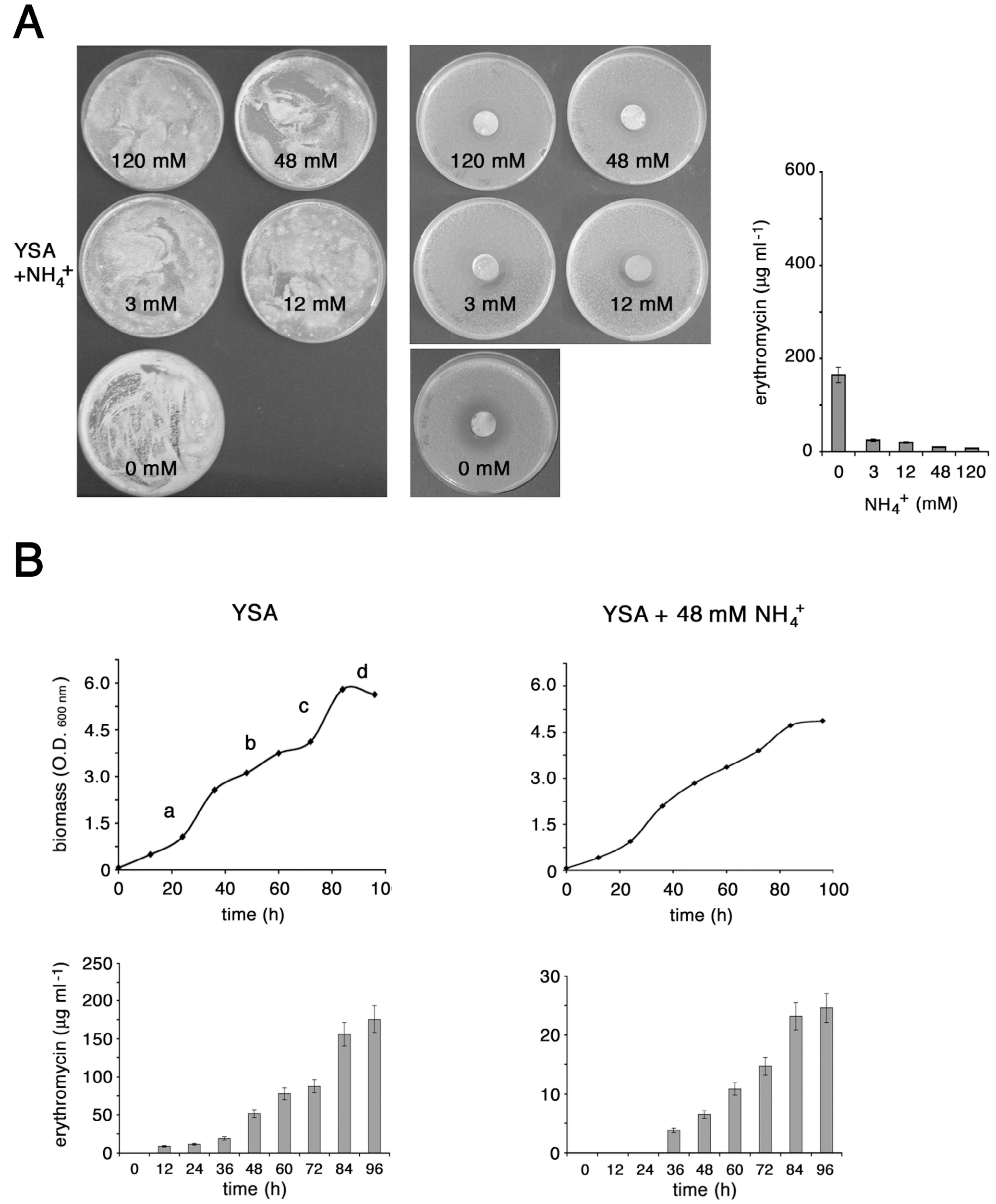

Figure S2

Effects of ammonium on *S. erythraea* pigmentation and erythromycin production in solid media. *S. erythraea* NRRL2338 was grown for 7 days on YSA medium supplemented with increasing ammonium (A) concentrations as indicated. The effects of the medium supplements on pigmentation (left) and on erythromycin production, which was evaluated by microbiological assays using *M. luteus* as a tester strain (center and right), are shown. In the right panels, data are shown as mean ± standard deviation of triplicate samples in representative experiments. Similar results were obtained in three independent experiments. (B) Effects of ammonium on erythromycin production in liquid media. *S. erythraea* NRRL2338 was grown in YS broth supplemented with 48 mM ammonium (right) or in YS broth without supplement. Mycelial biomass (upper panels) and erythromycin production that was evaluated by TLC (lower panels) are shown. Data are shown as mean ± standard deviation of three independent experiments.

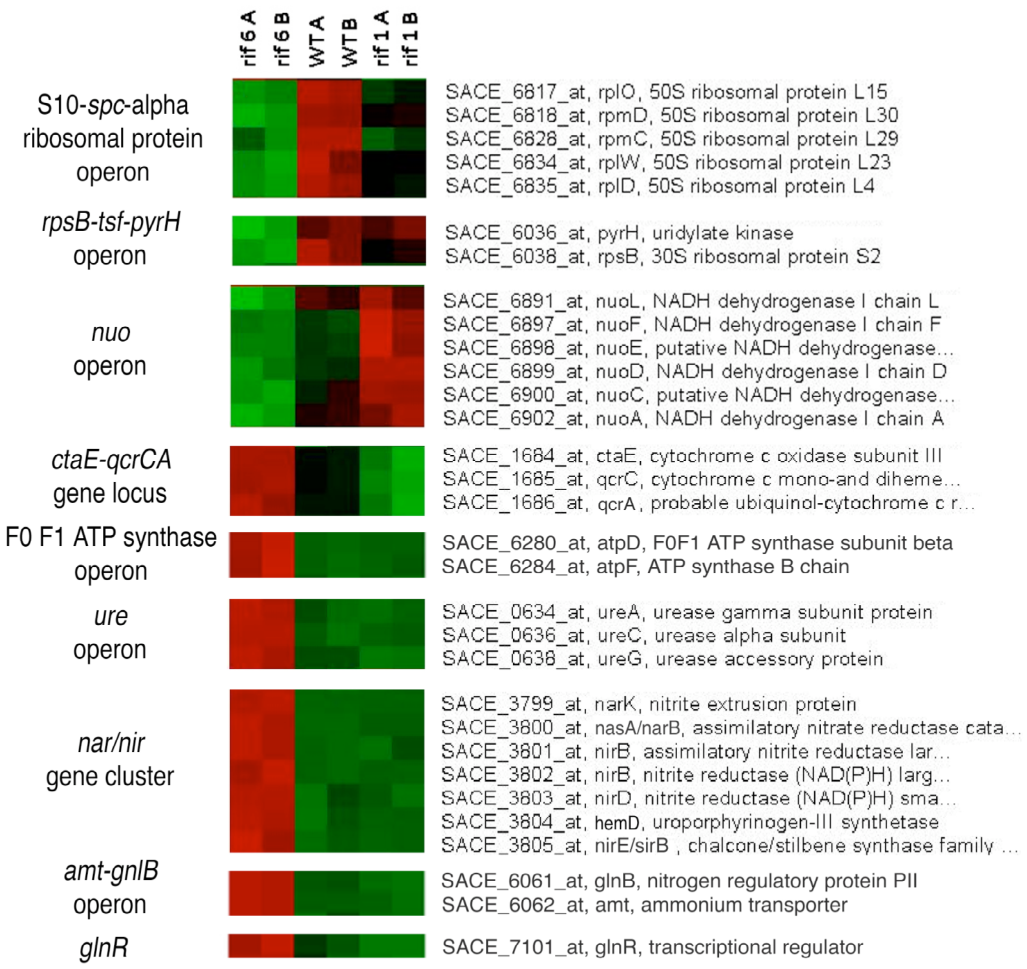

Figure S3

Members of known or putative operons showing similar expression profile. 26 out of 198 phase **a** differentially expressed genes are members of known or putative operons. Expression profile of SACE_7101 coding for the nitrogen regulatory protein GlnR is also shown.

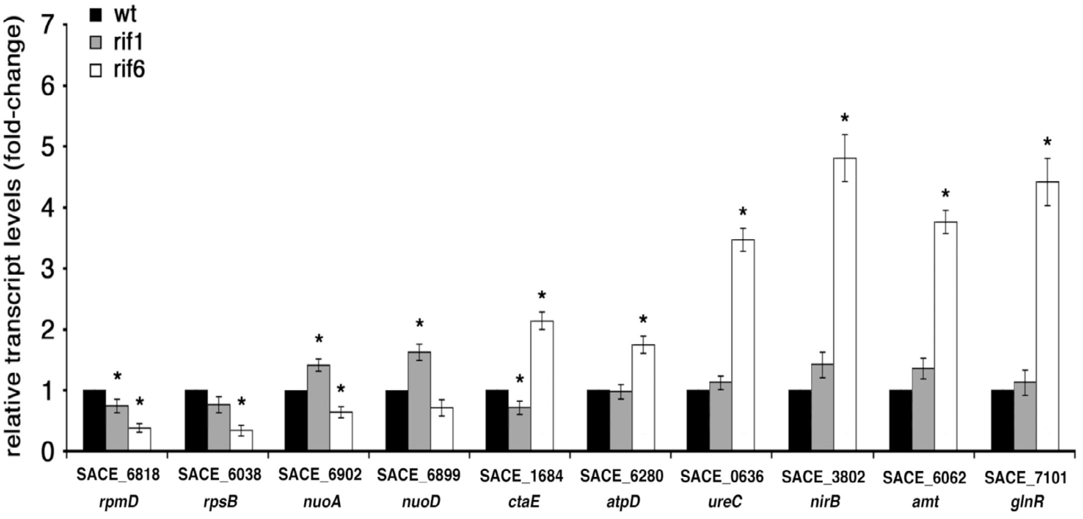

Figure S4

Semi-quantitative analysis of transcripts of genes located in known or putative operons by RT real time PCR. The RNAs were extracted from *S. erythraea* NRRL2338 and *rif* derivatives rif1 and rif6 grown in R3/1 medium up to phase **a**. Results were normalized to 16S rRNA levels. Transcript levels of *S. erythraea* NRRL2338 were arbitrarily given a value of 1. Data are shown as mean ± standard deviation from three independent experiments, each with triplicate samples, using distinct cDNA preparations for each RNA sample. The Student's t-test was used for statistical analysis. Statistically significant differences between values from *S. erythraea* NRRL2338 and *rif* mutants (asterisks) are declared at a p value <0.05.
